# Targeting Folate Receptors for the Detection and Selective Treatment of Cancer: Advanced Precision Oncology in Practice

**DOI:** 10.7150/ijbs.139733

**Published:** 2026-07-30

**Authors:** Michal Stark, Yehuda G. Assaraf

**Affiliations:** The Fred Wyszkowski Cancer Research Laboratory, Faculty of Biology, The Technion-Israel Institute of Technology, Haifa 3200003, Israel.

**Keywords:** folate receptor, cancer detection, selective targeting, tumor associated macrophages

## Abstract

The modern era of precision oncology strives to identify novel and selective druggable targets that, on the one hand, achieve efficacious anti-tumor activity, and on the other hand inflict minimal adverse effects to healthy tissues. The realization that both the tumor microenvironment and the tumor immune microenvironment (TIME) modulate tumor progression and response to chemotherapeutics, has pushed tumor-associated immune cells to the forefront as *bona fide* druggable targets. Based on their unique overexpression pattern on the cell surface of specific human malignancies and suppressor immune cells, folate receptors FRα and FRβ constitute optimal facilitators of noninvasive cancer detection and localization, as well as efficacious selective delivery of potent therapeutic payloads. Along this vein, this review highlights recent advanced strategies utilizing FR-targeting for precise tumor and metastatic lesion localization for guided surgical precision resection, potent anti-cancer efficacy and reprogramming of the suppressive TIME. Notably, these activities were accomplished with minimal side effects to healthy tissues. AZD5335, a recent clinically tested FRα-targeted antibody-drug conjugate carrying a topoisomerase I inhibitor payload, exceeded its predecessors by demonstrating remarkable efficacy against low FRα-expressing tumors as well. Thus, FRs emerge as selective and promising targets for advanced precision oncology of various malignancies including those displaying chemoresistance.

## 1. Introduction

One carbon metabolism, and specifically the folate cycle, provides one carbon units for the vital *de novo* biosynthesis of DNA (purine nucleotides and thymidylate) and amino acids (glycine and methionine), as well as for various cellular methylation reactions (DNA, RNA, histones, phospholipids and neurotransmitters) 1,2. As such, folates (vitamin B9) are imperative for normal fetal development 3,4 and cellular health 5, however, they are also essential for carcinogenesis and cancer progression 6,7. While folate cofactors are crucial for cellular functions, most eukaryotic cells lack the capability for *de novo* folate biosynthesis 1. Hence, several transport routes mediate the uptake of folates into cells 8. These include: 1) The predominant and ubiquitously expressed reduced folate carrier (RFC, *SLC19A1*) 9, 2) The principal intestinal folate absorption transporter, known as the proton-coupled folate transporter (PCFT, *SLC46A1*) 10, and 3) The glycosyl-phosphatidylinositol (GPI)-anchored membrane and soluble α, β and γ folate receptors (FRs) 11. Folic acid (FA) is a poor substrate of RFC (*Km* ~ 200 µM) which efficiently takes up the predominant blood circulating folate 5-methyltetrahydrofolate (5-MTHF) and other reduced folates with much higher affinity (*Km* ~ 1 µM) 12,13. PCFT functions as a proton-driven folate influx transporter. As such, PCFT displays comparable *Km* values (~ 1.5 µM) for FA and 5-MTHF at an acidic pH of 5.5, 14,15, whereas its affinity for FA markedly drops to 56 µM at pH 7.5 15. In comparison, at physiological pH, the acceptable dissociation constant (*Kd*) values for FA binding to FRα and FRβ reveal a high-affinity interaction in the range of 0.1-0.4 nM and 1-3 nM, respectively 16-19. These receptors exhibit slightly lower affinity towards 5-MTHF, i.e., *Kd =* 2-3 nM 16. Their soluble counterpart FRγ displayed *Kd* values of 5 and 2.5 nM for FA and 5-MTHF, respectively 20. It should be emphasized that these measured *Kd* values may vary in different reports based on both the distinct assays employed, like the gold standard isothermal titration calorimetry (ITC) 19 or competition assays 21, as well as the variable conditions used like pH, temperature, ionic strength and cell types. Notably, in studies using ITC measurements in which *Kd* values as low as 10 pM were reported 19, one has to take caution as the lower end of sensitivity of ITC is ~ 1 nM 22. Under acidic pH, the *Kd* of FRα for FA increases to ~ 20 nM 19, thereby allowing the release of FA within lysosomes.

Apart from their high affinity for FA, membrane bound FRs have unique tissue expression patterns. While FRα is often overexpressed in various malignancies 23, predicting dismal prognosis 24,25, FRβ is highly expressed by suppressive tumor-associated immune cells 26-29. For these reasons, FRs have been exploited for decades for targeted delivery of FA-conjugated anticancer drugs 30,31. The normal plasma concentration of circulating folates is at a low range of 10-40 nM 32,33. However, the specialized membrane folate transport routes and, most importantly, folate-polyglutamylation catalyzed by folylpoly-γ-glutamate synthetase (FPGS) 1, achieve an intracellular folate pool of 0.5-1 µM 34-36. Conversely, the serum folate concentration in mice used for *in vivo* experiments, is much higher than in humans, attaining up to 800 nM 37-39. Hence, the large difference between the serum folate concentration in men and xenograft murine models can markedly diminish the efficacy of folate-targeted experimental therapeutics in the preclinical setting 39. Specifically, the high concentrations of folates found in mouse serum readily compete with the tested agent on binding to FRs, thereby markedly compromising its cytotoxic efficacy 39. It has been established during the 1990's that mice should be fed with a folate-deficient diet before testing FR-targeted agents 37. Indeed, following feeding of mice with a folate-deficient chow, their serum folate concentration was reduced to 20-70 nM 37-39, thus being clinically representative of the human plasma folate concentration range.

In the present review, we summarize recent novel strategies exploiting FRs for the detection and targeted treatment of various malignancies, as well as the reprograming of the tumor immune microenvironment (TIME) 40. However, one should emphasize that most of the discussed results that were obtained in xenograft murine models, are apparently an underestimation, since almost all of the studies did not use a folate-deficient diet.

## 2. FRα in cancer detection

### 2.1. Fluorescence guided surgery with OTL38

The successful eradication of solid tumors primarily relies on the complete resection of the cancerous tissue. Various publications have validated the correlation between gross-total resection and prolonged survival 41-45. However, the “complete” resection based on a surgeon's assessment can be underachieved. This was demonstrated in a post-trial *ad hoc* analysis of a phase III randomized clinical trial, where 40% of advanced stage ovarian cancer (OC) patients (n = 627) displayed a residual disease (RD) >1 cm post optimal surgical cytoreduction 46. Consistently, an independent study also reported that 49% of advanced OC patients (n = 117) presented with RD >1 cm following optimal primary cytoreduction 47. In this respect, the gold standard for evaluating surgical margins is histopathological examination, which is restricted to examining a small portion of the resected surface, and requires a relatively long processing time of 2-5 days 48,49, frequently resulting in the need for re**-**excision 50.

Human FRα displays low baseline expression levels in normal tissues (0.3-3 pmol FR/mg solubilized membrane protein) except for the lungs and kidneys, which exhibit a mean of 7.8 and 14.4 pmol/mg, respectively 51. In contrast, OC is characterized by FRα overexpression with levels attaining a mean of ~ 35 and ~ 45 pmol/mg in 100% of papillary serous cystadenocarcinoma and metastatic ovarian adenocarcinoma, respectively 51. This led to the development of an FRα-targeting tumor-specific fluorescence imaging agent named OTL38 (Pafolacianine, Cytalux^®^) for real-time fluorescence-guided surgery of cancer. OTL38 is a folate analog ligand (pteroyl moiety) conjugated to a S0456 dye which absorbs at 774-775 nm and emits at 794-796 nm 52,53. These wavelengths are within the near infra-red (NIR) region, which is ideal for *in vivo* imaging, as it can penetrate several cm of tissue with minimal light absorption by hemoglobin (<650 nm) and water (>900 nm) 54,55. Following intravenous administration, the OTL38 folate analog targets FRα overexpressing cells with ~ 1 nM binding affinity, cells then internalize this probe and emit fluorescence upon excitation with a NIR laser 52.

In a randomized controlled trial, Charlotte E.S. Hoogstins *et al.,* examined the tolerability and pharmacokinetics of OTL38 in healthy volunteers, determining the optimal dosage and imaging time window 56. The low dose of 0.025 mg/kg OTL38 was selected as optimal since it elicited a few mild treatment emergent adverse events (TEAEs) and achieved the highest tumor-to-background ratio (TBR). OTL38 was then administered to 12 OC patients, allowing the intraoperative *in situ* fluorescent detection of FRα-positive tumors and metastases. Remarkably, 77% (n = 62) of fluorescent-guided resected lesions were confirmed as malignancies by histopathology, with 29% of them being undetected by standard methods 56. Around fifty percent of the false positive resected lesions were lymph nodes containing activated macrophages that, as will be further detailed below, express FRβ. The latter was found to be bound by OTL38 with equal affinity to FRα 52. The rest of the false positive lesions included FRα-positive epithelial cells that are routinely resected during cytoreductive surgery. Two false negative lesions were found, representing ~ 3% of malignant lesions. This study represented the first successful use of tumor-specific NIR fluorescence-guided OC surgery 56.

The overwhelming promising results obtained with OTL38 in OC led to a phase II, multicenter, open-label trial of intraoperative molecular imaging (IMI) (NCT02317705) 57. A sensitivity of 98% was estimated for the correlation of detection among multiple lesions within a single patient, and at least one undetected lesion was identified by OTL38 in 48% of the patients (n = 14) 57. A phase III clinical trial soon followed, encompassing 11 centers and 109 FR-positive OC patients (NCT03180307) 58. OTL38 identified undetected cancer tissues in ~ 35% of patients, its OC detection sensitivity was 83%, complete R0 resection was achieved in 62.4% of patients, and its false-positive rate was 24.8% 58. Systematic reviews of clinical and preclinical studies assessing the use of OTL38 IMI in OC have been reported 59,60. OTL38 was approved by the US Food and Drug Administration (FDA) in 2021 for the IMI and detection of OC 61.

Since several studies demonstrated overexpression of FRα in lung adenocarcinoma 62-65, OTL38 was tested during surgery of dogs with primary lung tumors and compared to video-assisted thoracoscopic surgery (VATS) 53. Tumor margins were evaluated by immunohistochemistry (IHC) of formalin-fixed paraffin-embedded (FFPE) tissue sections and additionally tested for FRα expression. OTL38 fluorescence was not only consistent with the pathological results but also facilitated the identification of undiscovered positive lymph nodes harboring cancer cells as well as retained tumor margin from the VATS procedure. Following these promising results, Jane Keating *et al.,* conducted a proof-of-principle study on three patients with diagnosed pulmonary adenocarcinoma. The test subjects were administered 3 hours prior to imaging with an injection of 0.025 mg/kg OTL38 via the median cubital vein, with no TEAEs. Post-operative pathology confirmed negative margins 53.

Following a small-scale phase I trial for the IMI of pulmonary adenocarcinomas 66, a multi-institutional phase II clinical trial was conducted, evaluating the use of OTL38 for IMI in non-small cell lung cancer (NSCLC) patients (n = 92) (NCT02872701) 67. OTL38-based IMI allowed the discovery of wrongly classified positive margins (9%) and undetected malignant lesions (12%). The IMI also detected synchronous cancer lesions that included primary adenocarcinomas, metastatic papillary thyroid cancer and adenoid cystic carcinoma. False negative detection by IMI included pre-invasive lung lesions and inflamed tissues containing activated macrophages 67. In a phase III, 12-center trial (NCT04241315) 68, among 100 lung cancer patients, OTL38 IMI identified undetected primary nodules in 19% of patients, as well as undetectable synchronous cancers (adenocarcinomas and metastatic chordomas) in 8% of them. The cancer detection sensitivity was 76% with a 17% false-positive detection. IMI-guided detection in 29% of the patients resulted in a change of the scope of the surgical procedure 68. OTL38 was also shown to be useful and safe for IMI of pulmonary metastases in young adults 69.

Clinical trials assessing the use of OTL38 for the IMI of other cancers are ongoing. In this respect, NCT06235125 and NCT06915727 are evaluating the safety of OTL38 in children with confirmed diagnosis of osteosarcoma, synovial sarcoma, hepatoblastoma, rhabdomyosarcoma, Ewing sarcoma, Wilms tumor and other solid tumors. NCT07124351 for gastrointestinal malignancies, NCT07278986 for endometrial cancer, NCT06434909 for pancreatic cancer, NCT06511037 for peritoneal carcinomatosis as well as NCT07039526 for digestive tract and gynecological malignancies.

Since OTL38 is restricted to FRα-positive tumors and FRβ-positive macrophages, the group of the pioneer PS Low set out to design a tumor fluorescence imaging cocktail with a broader scope 70. Towards this end, they generated a fibroblast activation protein (FAP)-targeted NIR dye conjugate (i.e., FAP9-S0456) with a FAP-specificity IC_50_ value of 2 nM. Since this conjugate consists of the same fluorophore as OTL38, both probes exhibited comparable excitation and emission spectra, allowing their co-excitation with a single laser 70. FAP, a serine protease, is a cell surface, single pass transmembrane protein 71. It is expressed on various cells of the tumor microenvironment (TME), primarily on cancer-associated fibroblasts (CAFs) 71,72. CAFs constitute a major component of the TME 73, where they support carcinogenesis, tumor growth and metastasis 74-76. As such, they are good indicators for tumor localization, and their counterparts metastasis-associated fibroblasts (MAFs, mCAFs) are indicators of the localization of metastases 77,78. The combination of FAP9-S0456 and OTL38 was predicted to detect the vast majority of tumor types. The actual efficiency of this cocktail was assessed using murine models harboring tumor xenografts from multiple non FRα/FAP overexpressing malignant origins: murine breast cancer 4T1 cells, colon carcinoma CT26 cells and renal cortical adenocarcinoma RENCA cells, as well as human NSCLC A549 cells and pancreatic cancer MIA PaCa-2 cells 70. While the tumor fluorescence intensity was higher (2-3-fold) with the cocktail than with each probe alone, the TBR was hampered by the presence of FRα in healthy tissues. Thus, FAP9-S0456 had the highest TBR. When FRα-overexpressing human breast cancer MDA-MB-231 cells or FAP-overexpressing human glioblastoma U87MG cell xenografts were used, the cocktail-based tumor fluorescence did not exceed that of OTL38 or FAP9-S0456 alone, respectively. Taken together, the use of the cocktail may provide an advantage in ~ 50% of human cancers which neither overexpress FAP nor FRα 70.

### 2.2. The companion diagnostic tool for immunohistochemical detection of FRα

FRα-targeted therapy is based upon FRα overexpression by malignant cancers. In this respect, patients should be first scanned to validate their qualification for FRα-targeted therapy. Moreover, the use of a single detection system with a global FRα scoring method will help to minimize variations in interpreting FRα expression and allow to achieve diagnostic consistency. Along this vein, a test was developed to measure the expression of FRα in patient-derived FFPE blocks of epithelial OC. The VENTANA FOLR1 (FOLR1-2.1) RxDx Assay, developed by Ventana Medical Systems Inc and Roche Tissue Diagnostics, is a fully automated IHC assay, based on a mouse monoclonal anti-FOLR1 primary antibody (FOLR1-2.1) and an OptiView DAB IHC Detection Kit 79. Following the validation of the repeatability and reproducibility of the VENTANA FOLR1 assay, it was approved by the FDA as a companion diagnostic tool for the treatment with mirvetuximab soravtansine-gynx (MIRV, IMGN853, ELAHERE) 80. The latter is an FRα-targeted antibody-drug conjugate (ADC) on which we elaborate below. The VENTANA FOLR1 assay was utilized to screen and select patients as a participation criterion for a single-arm phase II study, named SORAYA (NCT04296890) 81,82 and a phase III randomized trial named MIRASOL (NCT04209855) 83. The trials evaluated the efficacy and safety of MIRV in advanced high-grade OC patients with high FRα expression, and compared MIRV to standard chemotherapy, respectively. Following these two pivotal studies, MIRV was approved by the FDA for the treatment of platinum-resistant epithelial ovarian cancer (PROC) 84,85. A retrospective study encompassing 425 OC patient specimens, primarily high-grade serous carcinoma, tested for FRα expression using the VENTANA FOLR1 assay 86. High FRα expression was found in 36.3% of the cases and was significantly associated with high-grade serous ovarian histology. Primary masses exhibited higher FRα levels than metastases (positive rates of 44.4% vs 32.5%, *p* = 0.02) 86. A second study was recently published with comparable results from a cohort of Chinese OC patients, where the VENTANA FOLR1 assay was used to analyze 313 samples for FRα expression 87. Three independent pathologists demonstrated a strong concordance (>97%) in the interpretation of the assay's results. A total of 40.9% of cases presented high FRα levels, and primary masses exhibited higher FRα levels than metastases (positive rates of 44.2% vs 32.2%, *p* = 0.04) 87. These results emphasize the requirement for standardized FRα testing before FRα-targeted treatment is administered and the advantage of a uniform FRα scoring method.

### 2.3. Serum FRα

While the VENTANA FOLR1 assay standardized patient specimen testing for FRα positivity, it is based on an invasive biopsy procedure. In search of a non-invasive, rapid and inexpensive procedure for assessing FRα positivity in tumors, researchers turned to the rapidly growing field of cancer serum biomarkers 88-91. The elevated expression of FRα in OC along with its ability to be secreted or shed from cells, despite its GPI-membrane-anchoring 92, has brought forth soluble FRα (sFRα) as a serum biomarker for the detection, diagnosis and monitoring of OC 89,93-95. Beyond OC detection, sFRα protein levels were shown to be associated with tumor burden, allowing the indirect monitoring of tumor shrinkage following treatment 94, and disease recurrence 95. One clinical trial, discussed in section 3.1.2.2, quantified the levels of circulating sFRα in patients during treatment, and demonstrated a transient peak in serum sFRα levels due to tumor cell death 96. Serum sFRα was also demonstrated to predict histological subgroups of medulloblastoma with sensitivity and specificity 97. FRα can be readily detected in serum samples using an ELISA and the variety of commercially available anti-FRα antibodies detailed below, as well as others which are being continuously developed 98.

## 3. FRα-targeted cancer therapeutics

### 3.1. Antibody-based cancer therapeutics

Antibodies have been utilized in biomedical research and diagnostics since the early 1940s, a use that has expanded with the introduction of monoclonal antibodies (mAbs) half a century ago by Kohler and Milstein 99. The increase in clinical demand along with technological advancements and the high specificity of mAbs, established them as a cornerstone in contemporary diagnostics and therapeutics. The FDA and the European Medicines Agency (EMA) have approved over 50 antibody-based therapeutics for oncology applications 100, in addition to chimeric antigen receptors (CARs) for T cell engineering 101,102. Antibody-based therapeutics include three main modalities, monospecific and bispecific antibodies 103, as well as ADCs 104,105. Monospecific antibodies are full-length immunoglobulins, typically IgG since it is the only isotype that is bound and protected by the neonatal Fc receptor (FcRn), leading to its long circulation half-life 106. Monospecific antibodies are designed to target cell surface antigens such as receptors for growth factors and differentiation marker glycoproteins that are overexpressed in solid tumors and immune cells, respectively 100. By targeting these ligands, monospecific antibodies elicit cancer cell death via several mechanisms 107,108: 1) Disruption of signaling; by binding cell surface receptors, monospecific antibodies directly block survival and proliferation signals by growth factors such as epidermal growth factor (EGF) and transforming growth factor alpha (TGF-α) 109. Furthermore, blocking the signaling of vascular endothelial growth factor (VEGF) abolishes angiogenesis and disrupts tumor blood supply. In this respect, bevacizumab (Avastin), used in the treatment of PROC, is a humanized mAb that targets VEGF itself and blocks its pro-tumoral signaling pathways 110,111. 2) Immunologic tumor destruction, including antibody-dependent cell-mediated cytotoxicity (ADCC) and antibody-dependent cell-mediated phagocytosis (ADCP). In these mechanisms, the antibody binds a cancer cell surface antigen via its variable regions, while its Fc region binds an Fcγ receptor (FcγR) on the surface of a leukocyte cell, such as a natural killer (NK) cell 112 or macrophage 113. The antibody recruits and activates effector immune cells which can either secrete proteolytic granules that induce cancer cell apoptosis 114, secrete cytokines that activate complement-dependent cell death (CDC) 115,116, or phagocytose the cancer cell 117. 3) Immune checkpoint blockade 118. Cancer cells often increase the expression of immune checkpoint surface proteins which allow them to evade host immune surveillance by suppressing the activation of immune cells, primarily cytotoxic T-lymphocytes (CTLs). Hence, targeted blocking of these checkpoint molecules by antibodies eliminate the immune suppression, thereby restoring the activation of the immune system against cancer cells 119.

#### 3.1.1. Anti-FRα antibodies

During the 1980s three cornerstone anti-FRα mAbs were raised against human ovarian carcinoma and choriocarcinoma using hybridoma technology, MOv18 and MOv19 120,121 and LK26 122.

##### 3.1.1.1. Farletuzumab (MORAb-003)

The murine LK26 mAb exhibited a *Kd* of 7 nM for native FRα and reduced the growth of SKOV3 cell xenografts in nude mice by ~ 62% compared to control IgG (*p* < 0.05) 123. For clinical application purposes, this antibody had to be humanized and preclinically evaluated. To this end, optimization of humanized LK26 mAb was performed using a whole cell genetic evolution platform, i.e., *morphogenics* technology 124. The latter platform has proven highly successful in generating antibody-producing cell lines which secrete antibodies with superb binding affinities as well as enhanced titers that are suitable for scalable manufacturing. Thus, this *morphogenics* technology platform yielded farletuzumab (MORAb-003), a high affinity (*Kd* = 2.2 nM) anti-FRα IgG mAb 123. With the use of FRα-expressing cell lines and human serum, Wolfgang Ebel *et al.,* demonstrated the ability of farletuzumab to induce CDC resulting in up to 98% cytotoxicity *in vitro*, compared to negligible toxicity by farletuzumab or serum alone. Moreover, by using a combination of farletuzumab and peripheral blood mononuclear cells (PBMCs) from healthy donors, the authors demonstrated the *in vitro* activation of ADCC, resulting in >70% cytotoxicity in FRα-positive cells vs. no toxicity in FRα-negative cells. Toxicology studies in female cynomolgus macaque monkeys receiving intravenous infusions of farletuzumab, revealed a very long antibody half-life of ~ 250 hours, with measurable serum levels up to 4 weeks following last infusion. Importantly, almost no anti-human antibodies were developed in response to the administration of farletuzumab 123.

A phase I study of farletuzumab was conducted with 25 PROC patients who previously underwent ≥3 lines of treatment (NCT00428766) 125. Dose escalation experiments attained 400 mg/m^2^ farletuzumab with no grade ≥3 TEAEs. Since no maximum tolerated dose (MTD) of farletuzumab was reached, a recommended phase II dose (RP2D) could not be determined. Tumor targeting was monitored via a radiolabeled farletuzumab in SW620 colorectal cancer xenograft mouse models and PROC patients via planar whole body gamma camera images 126. Following 4 weeks of farletuzumab treatment, 36% of patients had radiologically stable disease (SD) and 4 patients exhibited a ≤43% decrease in carcinoma-associated antigen CA-125 levels 125. Further clinical trials evaluating farletuzumab in combination therapy in OC patients did not show any improvement in treatment outcome 127-129. This suggests that the farletuzumab-dependent ADCC and CDC effects were not recapitulated *in vivo*, possibly due to the immune-suppressive TME generated following platinum treatment of OC 130,131. Thus, the high specificity and affinity of farletuzumab along with its safety were exploited for the design of the FRα-targeted ADC MORAb-202 132, which is discussed below.

##### 3.1.1.2. MOv18

Although FDA approved mAbs for cancer therapy have all been of the IgG isotype 108, the chimeric IgE MOv18 antibody (i.e., consisting of murine variable and human constant regions) was found to exhibit greater efficacy for a longer time against a murine xenograft model of OC compared to the IgG isotype 133. While IgE antibodies are established mediators of histamine degranulation-based allergic reactions, their ability to facilitate tumor destruction has gained much interest in the past two decades 134, leading to AllergoOncology, an evolving interdisciplinary new field encompassing allergy, immunology and oncology 135,136. IgE has the highest affinity to its cognate receptors of all immunoglobulins 134,137 and it has no known inhibitory Fc receptors such as the FcγRIIb, which potentiates immunosuppression in the TME 138. The development of IgE-mediated cancer therapy has not reached its full potential since the expression of FcεRI, the high-affinity receptor of IgE, is restricted to mast cells and basophils in mice, hindering preclinical evaluation. In humans, FcεRI is also expressed by dendritic cells (DCs) and monocytes, thus preclinical testing of IgE has been limited to *in vitro* experiments using donor-derived DCs and monocytes 139. Unlike IgG, IgE does not elicit ADCC *in vitro* 133,140. However, when accompanied by human-derived PBMCs, the chimeric IgE MOv18 antibody exhibited a more potent anti-tumor effect in a severe combined immunodeficient (SCID) xenograft mouse model of OC compared to its IgG counterpart 133. A subsequent study in a nude-mouse model of OC injected with PBMCs revealed, for the first time, that the IgE MOv18 chimeric antibody promoted ADCP through tumor infiltrating monocytes 141. This resulted in longer survival of the OC xenograft bearing mice, i.e., 40 ± 5.8 days vs. 22 ± 2.4 days, *p* = 0.012, for IgE and IgG MOv18, respectively. The chimeric IgE MOv18 antibody was able to elicit both ADCC and ADCP in a nude mouse OC model when injected in combination with U937 monocytes before and after interleukin-4 (IL-4) stimulation 142. U937 monocytes express both the IgE high affinity FcεRI and the low affinity FcεRII (CD23) receptors 143; with the expression of the latter being upregulated upon IL-4 stimulation. Hence, the authors demonstrated that ADCC was mediated via FcεRI, whereas FcεRII facilitated ADCP. This resulted in an increase in mean survival from 16 days to 27 days (*p* < 0.005), and from 17.5 days to 35 days (*p* < 0.0005), for MOv18 + untreated U937 and MOv18 + IL-4-treated U937 cells, respectively 142. Consistently, these findings were recapitulated in a nude mouse OC model using human-derived monocytes 144.

Further mechanistic insights were obtained by utilizing an immunocompetent syngeneic rat model harboring FRα-expressing lung adenocarcinoma metastases 145. The rat model better recapitulates the human IgE-FcεR system, since rat effector cells (*e.g.,* monocytes and macrophages) express trimeric FcεRI 146. Using chimeric IgE and IgG MOv18 antibodies, consisting of rat Fc sequences (i.e., rMOv18 IgE/IgG2b), the authors uncovered a role for IgE-mediated tumor-infiltrating macrophages in combating cancer *in vivo* 145.

As detailed in our recent review 147, M0 monocyte-differentiated quiescent cells undergo polarization to two distinct macrophage phenotypes, the pro-inflammatory/anti-tumoral M1 or the anti-inflammatory/pro-tumoral M2 phenotype. M1 macrophages directly eliminate malignant cells by phagocytosis and consequent autophagy, elicit cancer cell death by secreting cytokines such as tumor necrosis factor (TNF), as well as promote anti-tumor activities of other leukocytes. In stark contrast, M2 macrophages both abolish anti-tumor immune response and secrete pro-tumoral growth factors, thus representing a major component of the immune-suppressive TME 147. In the above syngeneic rat model, superior infiltration of rat macrophages into tumors was observed upon IgE rMOv18 treatment, compared to PBS (*p* = 0.003) and IgG rMOv18 (*p* = 0.03) 145. More importantly, IgE rMOv18 induced the highest percentage of tumor infiltrating CD80^+^ macrophages (*p* = 0.01), indicating they were of the M1-phenotype 148. Indeed, enhanced intracellular macrophage expression (*p* = 0.04) and secretion (*p* = 0.017) of TNF was measured upon IgE rMOv18 treatment 145. The M1 macrophage activation culminated in significantly lower lung metastases and tumor occupancy with rMOv18 IgE compared with rMOv18 IgG2b (<50%, *p* < 0.0001) or PBS (<30%, *p* < 0.0001). These results suggest that IgE antibodies can both polarize and recruit macrophages to attack cancer cells 145, confirming recent studies demonstrating the ability of IgE antibodies to reprogram M2 patient-derived macrophages 149-152; hence paving the way towards the possible clinical development and application of IgE antibodies to attack cancer and its metastasis.

Several clinical trials with chimeric MOv18 IgG have been conducted in OC patients with minor immunological effects 153. Hence, efforts were shifted towards the IgE antibody. A phase I, dose escalation trial was undertaken, using a chimeric first-in-class IgE Mov18 antibody in patients with FRα-positive solid tumors (NCT02546921) 154. Since a single patient, with circulating basophils at baseline, experienced anaphylaxis, patients were screened to ensure low risk of allergic toxicity by skin prick and basophil activation tests (BAT) 155. While the dose escalation study attained a dose of 12 mg MOv18 IgE, the MTD was not reached 154. The criteria for FRα-positivity was ≥5% of tumor cells with membrane positivity by IHC 156, hence, all final participants had gynecologic cancer (i.e., n = 21, 3 and 2 for OC, tubal carcinoma and endometrial cancer, respectively) 154. An increase in serum IL-6 levels was detected for up to 7 days following antibody administration, indicating activation of the immune system 157. However, no increase in TNF levels was observed, and only one patient exhibited signs of anti-tumor activity 154. This disappointing outcome could be a result of both an insufficient RFα-positivity threshold, as discussed below, or insufficient antibody dosing. In this respect, a phase Ib, open label expansion trial is ongoing (NCT06547840).

##### 3.1.1.3. MOv19

The original murine Mov19 mAb 120 underwent extensive development over the years 158, yielding a variety of active derivatives including a chimeric murine-human IgG mAb 159, a humanized mAb 160, humanized MOv19 fragment antigen-binding (Fab) fragment 161, MOv19 single-chain variable fragments (scFv) based CAR T cells 162-164, and the ADC MIRV 160.

#### 3.1.2. FR targeting ADCs

**Figure 1:**
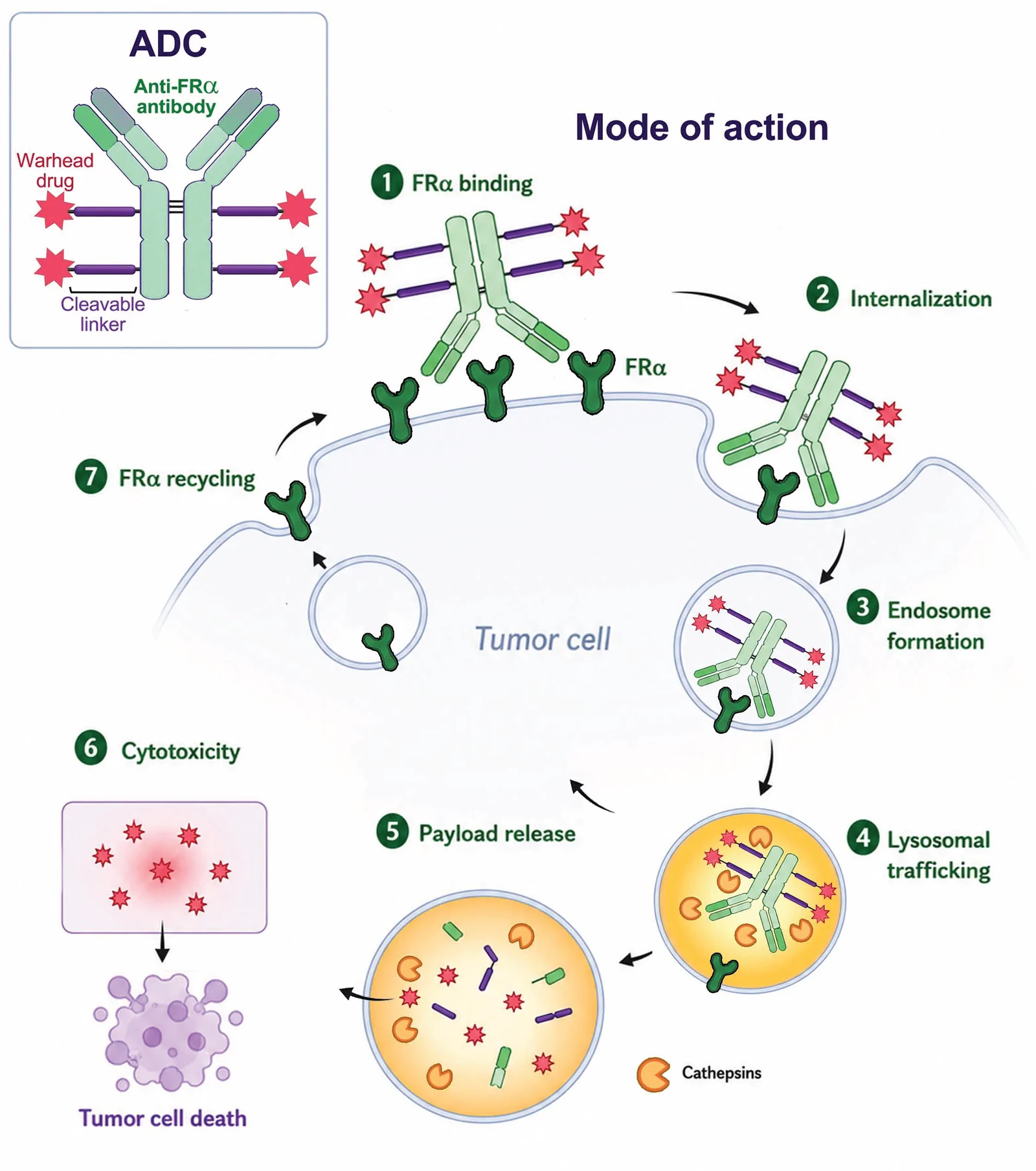
** Structure and mode of action of FRα-targeted antibody-drug conjugates (ADCs).** The ADC consists of an anti-FRα mAb conjugated to a cytotoxic drug, via a cleavable linker, with a drug:antibody ratio of ~ 4. 1) The ADC targets cancer cells via binding to FRα on the cell surface. 2) The binding of the mAb to FRα triggers receptor-mediated endocytosis. 3) The ADC is transported from the plasma membrane via an endosome which then fuses with a lysosome to form an endolysosome. 4) Within the acidic lysosome, FA dissociates from FRα which recycles to the plasma membrane (7). 5) Following reduction (i.e. cleavage) of the linker by glutathione or thiol reductase, and degradation of the mAb by lysosomal cathepsins, the drug payload becomes free to exit the lysosome into the cytosol. 6) The free drug exerts its cytotoxic activity, leading to cell death.

ADCs, referred to as "biological missiles” or 'magic bullets', consist of a mAb which is covalently attached via a chemical linker to a cytotoxic drug. ADCs are endowed with the important advantages of both high target specificity and potent killing, thus efficiently eliminating cancer cells with little off-target adverse effects 104,105. Unlike anti-FRα antibodies for IHC on fixed tissues, antibodies for ADC need to bind FRα in its native conformation and facilitate receptor-mediated endocytosis (RME) for the internalization of the cytotoxic drug conjugate. A scheme illustrating the mode of action of FRα-targeted ADCs is depicted in Figure 1.

##### 3.1.2.1. Mirvetuximab soravtansine-gynx

The FDA-approved ADC MIRV is an anti-FRα antibody conjugated through an *N*-succinimidyl 4-(2-pyridyldithio)-2-sulfobutanoate (sulfo-SPDB) linker to an *N*^2′^-deacetyl-*N*^2′^-(4-mercapto-4-methyl-1-oxopentyl)-maytansine (DM4) at a drug to antibody ratio (DAR) of ~ 4 160. During the development of MIRV, >100 murine anti-FRα antibodies were tested. The murine antibody-drug conjugates which exhibited the highest activity *in vitro* underwent humanization and were further tested *in vivo* using mice harboring highly FRα-positive KB xenografts. The *in vivo* experiments highlighted the superiority of one antibody, i.e., M9346A, the humanized derivative of the established anti-FRα antibody MOv19 158. Next, the cytotoxic activity of four linker/maytansinoid combinations of M9346A conjugates were compared using xenografts with varying FRα expression levels 160. For comparison, the mean FRα antibody-binding sites/cell in the xenografts' source cell lines were 4.5 × 10^6^, 1.3 × 10^6^ and 4.8 × 10^4^ for KB, IGROV-1 and OVCAR-3 cells, respectively. The highly hindered disulfide hydrophilic linker sulfo-SPDB 165 was better than a thioether-based linker, as well as better than both a hindered and a highly hindered disulfide linker 166. The M9346A-sulfo-SPDB-DM4 conjugate, i.e., MIRV, elicited the strongest anti-tumor effect, resulting in extended survival time of the murine models, especially towards the low FRα-presenting xenografts 160. The specificity of the conjugate was further verified *in vitro* by competition assays using saturating excess of the M9346A antibody. While MIRV exhibited sub-nM growth inhibition IC_50_ values in different highly FRα-positive cell lines, its cytotoxic activity decreased by >10-fold in the presence of the free antibody. However, when MIRV was tested against relatively low FRα expressing cell lines (i.e., ≤10^5^ FRα antibody-binding sites/cell), its IC_50_ values of 1-10 nM were not affected by the excess of free antibody. To explore the basis underlying the decreased sensitivity of the low FRα expressing cell lines to MIRV, the authors tested the intracellular processing capabilities of the cell lines 160. Maytansinoids are naturally occurring heterocyclic compounds that bind to β-tubulin in the α, β -tubulin heterodimer. Since their binding site is in the interface between tubulin heterodimers, maytansinoids hinder microtubule polymerization, resulting in microtubule destabilization, consequent mitotic arrest and cell death 167-169. When internalized, the DM4 maytansine conjugate is processed in the lysosomes, resulting in several active metabolites 168. Following incubation of the various cells with radiolabeled MIRV, and identification of the MIRV catabolites by HPLC, the authors concluded that the amount of processed active metabolites in the cells was proportionate to the cell surface antigen number 160. For example, KB cells processed 2.2 pmol/10^6^ cells, while OVCAR-3 cells, that express ~ 100-fold less surface FRα, processed only 0.02 pmol/10^6^ cells. Finally, the *in vivo* anti-tumor efficacy of MIRV was tested in murine models harboring NSCLC patient-derived xenografts (PDXs), mimicking clinically relevant cell surface FRα expression. MIRV treatment resulted in a substantial regression in tumor growth 160.

MIRV was further tested against biologically aggressive, type II, endometrioid and uterine serous carcinomas (USC) 170. First, FRα expression was evaluated via IHC in a retrospective cohort of 70 USC specimens. This revealed that 41% of the specimens exhibited moderate to high FRα expression, suggesting that this gynecological cancer should respond well to MIRV. Second, 20 primary cell lines were established from freshly derived endometrioid and USC tumor biopsies and evaluated for FRα expression using flow cytometry quantification. Moderate to high FRα expression was found in 22% and 27% of endometrioid and USC cell lines, respectively. These cell lines were then used in cytotoxicity assays, comparing MIRV to non-targeting ADC control and the free M9346A antibody. In FRα-overexpressing endometrioid cells, MIRV elicited 10-14-fold higher cytotoxicity than the ADC control (*p* < 0.01), while no such difference was observed in low-FRα expressing cells. Moreover, the free antibody had no detectable anti-tumor activity *in vitro*. In FRα-overexpressing USC cells, the impact of MIRV was also substantial but less prominent being 2.7-4.6-fold over the ADC control (*p* < 0.05). Interestingly, by using a co-culture of low- and high-FRα expressing endometrioid cells, the authors demonstrated that MIRV has a strong *in vitro* bystander effect, eliciting a 10-fold increase in cytotoxicity towards low FRα expressing cells (*p* < 0.01). Finally, the anti-tumor activity of MIRV was evaluated *in vivo*, using PDX murine models of both endometrioid and USC high-FRα expressing cells. Consistently, overall survival (OS) was significantly longer in MIRV-treated mice compared to controls (*p* < 0.001). Remarkably, the endometrioid-derived tumors were completely eradicated by MIRV, resulting in the survival of all mice at the end of evaluation (78 days) 170. Hence, endometrial cancer patients, and to a lesser extent USC patients, could apparently benefit from FRα-targeted MIRV treatment.

The first-in-human phase I clinical evaluation of MIRV as a monotherapy included 44 patients presenting with refractory tumors, i.e., OC >> endometrial cancer > renal cell cancer > NSCLC > cervical cancer, considered as FRα-positive (NCT01609556) 171. Two OC patients achieved confirmed partial response (PR), whereas SD was observed in 22 patients. Furthermore, 5 patients displayed a ≥50% reduction in the serum marker CA-125 for >28 days, defined as a CA-125 response 172. The adjusted ideal body weight (AIBW) of 6 mg/kg MIRV was chosen as the RP2D based on safety and activity 171. Taken together, the overall clinical benefit rate, i.e., PR + SD for ≥4 months + CA-125 response, was 23%. However, in this trial, FRα expression was not validated prior to treatment. Thus, in an expansion of the above phase I trial, FRα expression was a prerequisite, and the threshold for FRα positivity was defined as ≥25% of tumor staining at ≥2+ intensity by IHC. However, the overall clinical benefit rate remained 22% 173. These consistent findings suggested that a higher percent of FRα positive cells are required to achieve a beneficial cytotoxic effect by MIRV.

Advanced-stage epithelial OC is treated by surgery and platinum-based chemotherapy, which together achieve good initial results. However, most patients (~ 80%) relapse within several years and display platinum-resistant tumors. If disease progression occurs within 6 months of the last platinum-based regimen, the cancer is regarded as PROC 174. PROC is currently treated by non-platinum single agent (NPSA) chemotherapy (primarily paclitaxel, pegylated liposomal doxorubicin, or topotecan) alone or in combination with bevacizumab. A phase III FORWARD I trial, compared MIRV to other NPSAs in >300 patients with FRα-positive PROC 175. The threshold for FRα positivity by IHC was defined as ≥50% of tumor cells with any visible FRα membrane staining at ≤ ×10 microscope objective. While the required percent of FRα positive cells was higher than before, the ambiguous scoring of the level of FRα expression could present an impediment for successful MIRV therapy. Indeed, while some outcome parameters favored MIRV over the other chemotherapy agents, no statistically significant results were obtained 175.

The phase II SORAYA study utilized the VENTANA FOLR1 assay, to screen PROC patients according to their FRα expression status 81,82. In this study, the threshold for FRα positivity was defined as ≥75% of tumor cells exhibiting ≥2+ level of membrane staining intensity using the uniform grading score of the VENTANA FOLR1 assay. Another recruitment criterion was that patients already underwent 1-3 lines of therapy, one of which was bevacizumab. The clinical trial included 105 PROC patients, of which 5 achieved complete response (CR) and 29 achieved PR, increasing the objective response rate (ORR) above 30% (*p* < 0.0001). The highest ORR was obtained in patients previously treated by poly ADP-ribose polymerase inhibitor (PARPi), i.e., ORR 38.0% and 27.5% in patients who underwent prior PARPi treatment (n = 51) and those who did not (n = 54), respectively 82. Interestingly, serum sFR was proposed as a biomarker for PARPi resistance in OC patients, as high levels of FRα were associated with non-responders (*p* < 0.0026) 176. A reduction in tumor size occurred in 71.4% of patients, and the disease control rate (CR, PR, or SD ≥ 12 weeks) was 51.4% 81 vs. the previously reported 22.0% 173. Median OS was 15.0 months 82. This promising clinical trial was followed by the phase III randomized trial MIRASOL, which included 453 PROC patients from 21 countries 83,177. The same recruitment criteria were used as in the SORAYA study, except for the requirement for a previous bevacizumab treatment, and MIRV treatment outcome was compared to other NPSAs. The reported ORRs were 42.3% and 15.9% for the MIRV and the NPSAs groups, respectively (*p* < 0.001), resulting in longer OS times of the MIRV-treated patients, median, 16.46 months vs. 12.75 months (*p* = 0.005) 83. The SORAYA and MIRASOL studies underscored the importance of correct patient participation criteria and FRα-positivity scoring for efficient and beneficial treatment of PROC with MIRV, and established MIRV as a preferential therapy over standard NPSAs.

MIRV was further evaluated as a third-line, or greater, treatment of patients with recurrent platinum-sensitive ovarian cancer (PSOC) 178. The single-arm, phase II, PICCOLO trial (NCT05041257) used the same FRα positivity threshold as the SORAYA study 81 and required patients to have radiographic progressive disease (PD) following ≥2 lines of platinum-containing therapy. Initial results included an ORR of 51.9%, while OS was not mature at last follow-up. MIRV exhibited notable efficacy and tolerable safety 178. GLORIOSA, an ongoing phase III randomized, open-label trial (NCT05445778), will evaluate the combination of MIRV with bevacizumab vs. bevacizumab alone as maintenance therapy in high-FRα PSOC patients 179. The actively recruiting GLORIOSA study is planned to run through 2029.

##### 3.1.2.2. MORAb-202

A second FRα targeted ADC, designated MORAb-202 (farletuzumab ecteribulin, farletuzumab-[Mal-PEG2-Val-Cit-PAB-eribulin]), includes farletuzumab conjugated to the highly potent microtubule targeting agent (MTA) eribulin via a cathepsin cleavable valine-citrulline (Val-Cit) linker 180 at a DAR of 4 132. Eribulin mesylate (Halaven), used in the treatment of heavily pretreated metastatic breast cancer 181 and liposarcoma 182, binds β-tubulin at the microtubule plus-end and slows/prevents elongation leading to microtubule catastrophe 183. Importantly, while eribulin exerts functionally irreversible antimitotic effects, in addition to several non-mitotic anti-tumor effects such as tumor vascular remodeling and perfusion 184, it inflicts markedly less peripheral neuropathy compared to other MTAs, such as taxanes (*e.g.* paclitaxel) and *Vinca* alkaloids (*e.g.* vincristine) 185,186. MORAb-202 exhibited high specificity and potency during *in vitro* cytotoxicity assays, with superb IC_50_ values of 0.01-0.06 nM in high-FRα expressing cells as opposed to >100 nM in FRα-null cells 132. In moderate to low FRα expressing cell lines, the IC_50_ values were at the sub-nanomolar to nM range. *In vivo* evaluation of MORAb-202 using a moderate FRα expressing human NSCLC NCI-H2110 xenograft model, revealed that at a dose of 5 mg/kg MORAb-202 completely abolished tumor growth, thereby inducing a CR. Remarkably, at the end of the study (day 80), all mice were tumor-free with no effect on body weight. MORAb-202 was further evaluated using two PDX mouse models, the human NSCLC LXFA-737 and gastric cancer GA0055, both expressing moderate levels of FRα. Although CR was not achieved in all cases, a dramatic and durable tumor growth inhibition (TGI) was apparent 132. Notably, since these results were obtained following a single MORAb-202 dose, additional timely injections could extend the duration of the response. Indeed, in an extensive study with PDXs of poor prognosis gynecological cancer subtypes, the authors showed the contribution of additional dosing 187. Mice harboring low-FRα high-grade serous OC xenografts which exhibited PD after 85 days, received additional 3 weekly doses and achieved CR at least until day 120. Additional preclinical testing of MORAb-202 revealed a possible bystander effect both *in vitro* and *in vivo* 188. The cytotoxicity IC_50_ value of MORAb-202 in FRα-negative acute myeloid leukemia (AML) HL-60 cells dropped a 100-fold to 2.4 nM when co-cultured with high-FRα expressing IGROV-1 cells. In the OD-BRE-0631 PDX model of triple negative breast cancer (TNBC), MORAb-202 displayed a moderate anti-tumor effect which slowed tumor growth, resulting in a ~ 2.4-fold smaller tumor at day 31 (*p* < 0.0001) 188. This xenograft expresses low levels of FRα and is highly enriched in tumor stroma, both of which hinder targeted therapy 189. The stroma develops, in part, by the interstitial transformation of normal fibroblasts into CAFs. While the former cells express low levels of alpha-smooth muscle actin (α-SMA) and FAP, CAFs overexpress these proteins 75. Hence, using an anti-α-SMA antibody, the authors explored the status of the stroma in the OD-BRE-0631 xenograft mouse model following treatment with MORAb-202 188. The study showed that ablation of the CAF network was most profound in the vicinity of apoptotic tumor cells, presumably as a result of the diffusion and infiltration of released eribulin into neighboring tumor areas, resulting in a bystander killing effect. An additional TNBC PDX model was used, the high-FRα M-BRE-0563 model. In this model, MORAb-202 achieved a complete and lasting elimination of the tumors, up to the end of the experiment on day 60. Free eribulin, at an equivalent drug dose, had little to no anti-tumor effect, highlighting the advantage of targeted therapy. The serum levels of sFRα protein were monitored during MORAb-202 treatment, as a marker for the successful eradication of the OC tumor. Serum sFRα levels peaked 1-2 days after the administration of MORAb-202, suggesting the release of FRα by dead tumor cells. sFRα levels in MORAb-202 treated mice decreased back to the initial levels, while they retained a steady increase in mock treated mice, indicating the increase in tumor size. Toxicology studies in cynomolgus monkeys established bone marrow myelosuppression as the primary MORAb-202-related TEAE 188. This off-target toxicity of MORAb-202 could stem from the instability of its linker *in vivo*, as was demonstrated by the reduction of its DAR from 4 to 3 during a 10-day incubation period in human and mouse sera *in vitro* 132, indicating the release of free eribulin into the bloodstream. At a dose range of 2-6 mg/kg, the myelosuppression was reversible with neutropenia being resolved by day 21 188. Elimination half-life of MORAb-202 in plasma was 135-178 hours. Hence, the suggested MTD was 6 mg/kg every 3 weeks.

A first-in-human phase I study of MORAb-202 was conducted in 22 patients with IHC-confirmed FRα-positive solid tumors who failed to respond to standard therapy (NCT03386942) 96. Fifty five percent of patients had OC and the rest harbored other carcinomas including breast, endometrial, NSCLC, and fallopian tube cancer. Patients received low doses of 0.3-1.2 mg/kg MORAb-202 once every three weeks. The vast majority of the patients (95%) suffered from either leukopenia or neutropenia, however, all hematological adverse events were of grade 1 or 2. There were seven grade 3 and 4 TEAEs which included primarily increased levels of alanine aminotransferase and γ-glutamyl transferase, indicating liver cell damage. The serum concentrations of circulating sFRα were measured in each patient before and after the first and second dosing cycles. A dose-dependent increase in serum sFRα levels was recorded for 24 h following MORAb-202 treatment, albeit the amount of serum sFRα returned to its baseline level when cycle 2 was initiated. A positive correlation was observed between normalized serum sFRα levels and tumor shrinkage (*p* = 0.029). One OC patient achieved CR, nine patients attained PR and eight presented a SD; these tumor responses were seen within the entire MORAb-202 dosing range and in all types of cancer 96. A follow up expansion study was conducted with an additional 15 NSCLC patients, where the criterion for FRα-positivity was ≥5% of tumor cells stained at any intensity by IHC 190. Unsurprisingly, this small study was completed with modest results. As was evident from the MIRV clinical trials, FRα-positivity for successful treatment with an FRα-targeted ADC requires ≥75% of tumor cells stained with ≥2+ intensity. A phase II open-label randomized study in >100 patients with platinum-resistant high-grade serous gynecological cancers has been recently completed (NCT05613088). MORAb-202 was compared to other NPSAs, and in both groups >80% of patients displayed PD. 12% of patients in the MORAb-202 arm are still being treated vs. 6% in the NPSA arm. Given these results, an additional clinical trial is ongoing (NCT04300556).

##### 3.1.2.3. STRO-002

Conventional ADCs are synthesized by conjugating the drug of interest to a lysine or cysteine on the antibody surface. However, since common antibodies contain >50 lysine and 8 cysteine residues in four pairs of interchain disulfide bonds, conventional synthesis results in a heterogeneous mixture of ADCs that vary qualitatively and quantitatively in conjugation sites. Hence, this conjugation produces ADCs with a range of DARs, which can affect solubility, stability and activity 191-193. To overcome these variabilities, methods have been developed for the synthesis of homogeneous ADCs 191-193.

Although only recently developed 194, STRO-002 (luveltamab tazevibulin), a homogeneous FRα-targeted ADC, has already been evaluated in six phase I/II/III clinical trials. However, due to strategic business considerations only two trials were executed through to completion (NCT03748186, NCT06238687). The homogeneity of STRO-002 was achieved by using a patented SP8166 anti-FRα antibody (Patent WO2019055931). SP8166, discovered by ribosome display technology, is the product of the H01 anti-FRα antibody, in which the artificial amino acid *para*-azidomethyl-L-phenylalanine 195 was incorporated at positions Y180 and F404 within the heavy chain sequence. This allowed for a site-specific conjugation with the commercially available click chemistry linker-warhead SC239, resulting in a robust and homogenous (>97%) DAR of 3.9 194. For comparison, during the synthesis of MIRV, the maytansinoid per antibody ratio varied from 3.3 to 5.0 160. SP8166 exhibited remarkable *Kd* values of 2.3-9.9 nM to the extracellular domain of the human and cynomolgus monkey FRα overexpressed in cell lines, with weak, non-saturating binding to FRβ 194. SC239 consists of a cathepsin cleavable Val-Cit linker and the hemiasterlin synthetic analogue 3-aminophenyl hemiasterlin (SC209). Hemiasterlin is a marine sponge cytotoxic tripeptide MTA, which induces mitotic arrest at sub-nanomolar concentrations 196. Its small size, high cytotoxic activity and zwitterionic structure make hemiasterlin a good drug payload for ADCs 197. Importantly, hemiasterlin analogues were shown to surmount P-glycoprotein (P-gp, ABCB1)-dependent multidrug resistance *in vitro* and* in vivo* 198, and total synthesis of hemiasterlin and its analogues has been previously achieved 199. *In vitro* tubulin polymerization assays revealed that SC209 is more potent as an inhibitor of tubulin polymerization than DM4, used in the MIRV ADC 194. STRO-002 exhibited 10-50-fold lower cytotoxic IC_50_ values than free SC209 in FRα expressing cell lines, further establishing the advantage of targeted therapy. The stability of STRO-002 was tested via its incubation in human and monkey sera *in vitro* as well as mouse serum *in vivo*, where STRO-002 maintained a DAR of 3.9 up to 20 days. For comparison, when MORAb-202 was incubated *in vitro* for 10 days in human and mouse sera, its DAR was reduced from 4 to 3 132. Following STRO-002 treatment, its stability was further demonstrated by the presence of free SC209 exclusively within the tumors of IGROV-1-xenograft bearing mice 194. This indicates the specific release of the drug from the ADC by cathepsin cleavage within the lysosomes of FRα-targeted cells, hence suggesting minimal future off-target untoward toxicity. To ensure that the conjugation did not hinder the specificity and affinity of SP8166 to cell surface FRα, cytotoxicity assays were undertaken using FRα-positive and null cell lines. STRO-002 exhibited IC_50_ values of ≤0.05 nM in human FRα-positive IGROV-1 and OVSAHO cells, values that increased by 68-2000-fold in the presence of excess free SP8166. No growth inhibition was observed in FRα-null A549 and Daudi cells up to 100 nM. As seen with the ADCs described above, when Daudi cells were co-incubated with IGROV-1 cells, the former displayed growth inhibition at 0.59 nM STRO-002, indicating a strong bystander effect. These results establish the high specificity and potency of STRO-002 *in vitro*. The levels of circulating sFRα, specifically its extracellular domain, were quantified in serum samples from OC patients and healthy individuals. The highest level of sFRα detected in healthy controls was 1.39 ng/ml, while OC patients exhibited up to 80.46 ng/ml. To validate that STRO-002 will retain its *in vivo* cytotoxic potency in the presence of such high levels of circulating sFRα, the authors conducted additional cytotoxicity assays supplemented with up to 100 ng/ml recombinantly-expressed FRα extracellular domain; surpassing the highest amount found in human serum. The IC_50_ values of STRO-002 were not affected by the vast excess of soluble protein. *In vivo* evaluation of STRO-002 included several stages: Firstly, the dose-dependent anti-tumor activity of STRO-002 was tested against established (∼ 150 mm^3^) IGROV-1 xenografts in SCID mice. Remarkably, at single STRO-002 dose concentrations of 10 and 15 mg/kg, tumor regression was evident beyond day 30 and 50, respectively, while a dose of 5 mg/kg achieved tumor stasis until day 26. OVCAR-3-based tumors, which in this study express 7-fold less surface FRα than IGROV-1, regressed up to day 40 following a dose of 5 mg/kg STRO-002 with a total TGI of 81%. Next, in nude mice bearing a more advanced OVCAR-3-based tumor (∼ 400 mm^3^), TGI following a single STRO-002 dose of 10 mg/kg, developed into tumor regression up to day 60. OV-90-based xenografts in SCID mice, which harbor ≤10^5^ FRα antibody-binding sites/cell, exhibited relative resistance to STRO-002 194; as was demonstrated with MIRV, ≤10^5^ FRα antibody-binding sites/cell are the lower limit of FRα-targeted therapies 160. In these resistant mouse models, a combination treatment of STRO-002 (5 mg/kg) with bevacizumab (5 mg/kg) achieved 96% TGI vs. STRO-002 (29% TGI, *p* < 0.0001) or bevacizumab (68% TGI, *p* < 0.0054) alone 194. These results demonstrated the ability of STRO-002 to perform well both as monotherapy and in combination therapy for the treatment of OC, depending on the expression level of FRα. Finally, treatment with STRO-002 was evaluated in endometrial carcinoma PDX models. PDXs (n = 20) with an IHC-based FRα expression scoring of 0-4 were chosen, and mice were treated with 10 mg/kg STRO-002 weekly. A TGI >50% was observed in 53% of all FRα positive PDXs, while the response rate increased to 85% (6/7) in the highest-FRα expressing PDX group, out of which 2 exhibited complete tumor regression until the end of the experiment (day 45) 194.

Following the successful and extensive preclinical study in OC and endometrial carcinoma mouse models, STRO-002 was further evaluated for the treatment of NSCLC 65. As stated above, non-squamous NSCLC tumors tend to overexpress FRα, and are hence considered good candidates for FRα-targeted therapy. In a study by Robert Yuan *et al.* 65*,* 29% of non-squamous NSCLC patient-derived specimens expressed moderate to high levels of FRα. NSCLC PDX mouse models were established and administered with 4 weekly doses of 10 mg/kg STRO-002; although this seems high in comparison to other ADCs, no body weight loss was observed following treatment. Of seven mice harboring PDX with ≥25% of FRα-positive tumor staining, six displayed tumor regression until study completion, one of which achieved CR. In contrast, of the six mice harboring PDX with <25% of FRα-positive tumor staining, one exhibited a SD and the rest progressed rapidly 65. These results demonstrate the robust anti-tumor activity of STRO-002 against high-FRα expressing tumors.

Immunogenic cell death (ICD) is a type of regulated cell death, defined by damage-associated molecular patterns (DAMPs) that facilitate the recognition of dying cells by the immune system 200. The three most common DAMPs associated with ICD are the extracellular release of the high mobility group box 1 (HMGB1) protein and adenosine triphosphate (ATP), or the cell surface exposure of calreticulin (CRT). These DAMPs attract and activate antigen-presenting cells (APCs), which initiate an inflammatory response 201. ICD of cancer cells has a therapeutic advantage, since the release of tumor-derived antigens in the presence of DAMPs promotes their cross-presentation by APCs. This can elicit innate, adaptive, immediate and prolonged immune responses via the activation of various leukocytes, further enhancing the initial anti-tumor effect 200.

It has been demonstrated that several MTAs can induce ICD 202-204. For example, monomethyl auristatin E (MMAE) 204, which is the antimitotic agent serving as the payload in the clinically approved ADC brentuximab vedotin (Adcetris) 205,206. In this respect, the ability of STRO-002 to induce ICD was explored 65. *In vitro* testing included both STRO-002 and the free SC209, and utilized the FRα-positive and FRα-negative human tumor cell lines KB and A549, respectively. Following treatment with both agents, KB cells exhibited the three canonical DAMPs associated with ICD, including >4-fold increase in released HMGB1 (*p* < 0.0001) and ~ 3-fold increase in surface expression of CRT (*p* < 0.0001). A549 cells responded similarly, albeit only to SC209, since the ADC could not target them. Next, the authors verified the actual induction of an immune response by STRO-002 and SC209 via co-culturing treated tumor cells with human derived PBMCs. Immune activation was validated by monitoring the expression levels of CD86 on monocytes/macrophages, as well as the phagocytosis of tumor cells by macrophages 65. CD86 is a critical costimulatory molecule that is rapidly upregulated on the surface of activated professional APCs, like macrophages and DCs 207,208, and participates in the activation of CTLs 209 and NKs 210. In accordance with the ICD observed following treatment of KB cells with STRO-002 and SC209, co-cultured monocytes from two different donors exhibited upregulation of surface CD86 and activation of tumor cell phagocytosis; both parameters were stronger in response to STRO-002 compared to SC209 65. In A549 cells, only high concentrations of SC209 elicited the upregulation of CD86 on the surface of monocytes, whereas no phagocytosis was observed. The ability of STRO-002 to induce ICD *in vivo* was evaluated using a syngeneic murine model, harboring murine colorectal cancer MC38 cell xenografts engineered to express human FRα (i.e., MC38-hFRα cells). Before challenging the mice with a subcutaneous injection of MC38-hFRα cells for xenograft establishment, the mice were “vaccinated” with pretreated MC38-hFRα cells. To this end, MC38-hFRα cells were exposed *in vitro* for 72 h to either SC209, STRO-002 or cisplatin (which does not induce ICD), achieving ~ 85%, ~ 40% and ~ 100% cell death, respectively. Treated cells (~ 10^6^), resuspended in PBS, were injected subcutaneously into the left flank of model mice. Fourteen days later, vaccinated and naïve mice were injected in the contralateral flank with ~ 0.5 × 10^6^ untreated MC38-hFRα cells, and monitored for tumor growth. Naïve mice (n = 45) exhibited a comparable tumor-rejection rate as those vaccinated with cisplatin-treated MC38-hFRα cells (n = 25), i.e., 17.8% and 13.3%, respectively. In contrast, in mice vaccinated with STRO-002-treated cells (n = 18), 83% remained tumor-free for 39 days. Vaccination with SC209-treated cells resulted in a similar tumor rejection rate, where 76% of mice (n = 25) remained tumor-free. This demonstrated for the first time the ability of a hemiasterlin derivative to induce robust ICD, resulting in prolonged anti-tumor immune activation. Examination of MC38-hFRα-based xenografts revealed a STRO-002-dependent TIME reprograming. Specifically, STRO-002 treatment induced an increase in tumor infiltrating monocytes (*p* < 0.0001), increased macrophage CD86 expression (*p* < 0.01) and activation of CTLs (*p* < 0.01) 65.

The treatment of NSCLC has been dramatically transformed with the introduction of immune checkpoint inhibitors (ICIs) 211-213. Since the anti-tumor activity of STRO-002 relies, in part, on the activation of an anti-tumor immune response, the research group of Helena Kiefel explored the possible benefit of combining STRO-002 with the FDA approved anti-PD-L1 antibody avelumab 65. Avelumab, approved for the treatment of metastatic Merkel cell carcinoma and urothelial carcinoma 214,215, exerts its activity by blocking the interaction between the T-cell receptor programmed cell death protein 1 (PD-1) and its tumor surface ligand PD-L1, thereby circumventing immunosuppression 216. When treating MC38-hFRα tumor (~ 150 mm^3^) bearing mice, STRO-002 and avelumab monotherapies achieved 44% and 67% TGI, respectively, whereas the combination of the two resulted in a staggering 94% TGI with 40% CR rates 65. Five weeks after treatment, complete responders were re-challenged with either parental MC38 or MC38-hFRα cells (10^6^), and monitored for tumor growth for an additional 60 days. Owing to the robust immediate and prolonged immune responses elicited by the combination therapy of STRO-002 and avelumab, mice injected with MC38-hFRα cells (n = 7) completely rejected the re-challenge and remained tumor free. Since the vaccination occurred in the presence of MC38 antigens as well, 3 of 7 mice re-challenged with FRα-null MC38 cells also remained tumor free. Furthermore, while each single agent had no effect on the number of tumor infiltrating CTLs, the combination of the two resulted in an increased density of tumor CTLs 65. Thus, the combination of STRO-002 and avelumab appears to elicit a potent and robust anti-tumor immunity that should prevent the recurrence of the original tumor as well as prevent metastases.

A phase I, dose escalation study of STRO-002 has been completed in PROC patients without prior selection for FRα expression (NCT03748186). STRO-002 was administered once every 3 weeks, at a dose range of 0.5 to 6.4 mg/kg. Initial results showed that doses ≥ 2.9 mg/kg were clinically active, with a clinical benefit rate of 48%, i.e., 17% of evaluable patients (n = 29) achieving PR and 31% patients presenting a SD. A >50% reduction in serum CA-125 levels was found in 64% of patients. A percentage of the clinical response remained durable, with 36% and 24% for >16 and >24 weeks, respectively. The vast majority (88%) of TEAEs were of grades 1-2, however reversible grade 3-4 neutropenia occurred in 39% of the patients. Dose-limiting toxicity was reported at the highest doses of STRO-002 (6.0-6.4 mg/kg) 217. In an expansion follow up study, participants included 44 advanced OC patients with both platinum resistance and sensitivity. FRα expression was evaluated via the VENTANA FOLR1 assay, and patients with >25% of tumor positivity (n = 35) were evaluated. STRO-002 was administered at two doses of either 4.3 mg/kg or 5.2 mg/kg, and elicited grade ≥3 neutropenia in 70.5% of the participants. However, dose delay due to TEAEs occurred in 80% of the patients, with 3 patient withdrawals, suggesting lower doses of STRO-002 should be considered. An ORR of 37.5%, with a median response duration of 5.5 months were reported 218. Hence, further clinical trials are warranted.

##### 3.1.2.4. AZD5335

AZD5335 is a novel FRα-targeted ADC that differs from the previously described ADCs in its drug payload; instead of an MTA, AZD5335 consists of the topoisomerase-1 inhibitor (TOP1i) (4-NH_2_)-Exatecan (AZ14170132) 219. The latter is an ADC-oriented derivative of exatecan (DX-8951f) 220, which displayed superior cytotoxicity against various tumor cell lines and PDXs compared to classic TOP1is such as topotecan and the active metabolite of irinotecan, SN-38 221. Moreover, unlike topotecan and SN-38, the water-soluble exatecan was shown to surmount multiple mechanisms of multidrug resistance, including those based on P-gp and breast cancer resistance protein (BCRP, ABCG2) 222. TOP1 is an essential enzyme that catalyzes the underwinding of DNA supercoils during DNA replication and transcription, hence the trapping of TOP1 in a ternary complex with DNA by TOP1is results in cell death 223. AZD5335 consists of the patented anti-FRα mAb INT-019 (PCT WO 2023/169896 A1), generated by classical hybridoma technology, which displayed high affinity to both human and cynomolgus monkey FRα (*Kd* = 15.74 nM and 36.86 nM, respectively), and a ~ 250-fold lower affinity to murine FRα and human FRβ 219. The efficient FRα-dependent cellular internalization and lysosomal trafficking of INT-019 was validated using a fluorescent conjugate. The TOP1i payload was conjugated to native interchain cysteines within the INT-019 mAB, via a protease-cleavable maleimide-PEG8-valine-alanine linker, with a DAR of ~ 8. A 7-day *in vitro* incubation period in human, monkey and murine sera resulted in <4% deconjugation of AZD5335, demonstrating its stability. Moreover, following conjugation, AZD5335 maintained the original affinity values of its parental INT-019 mAB. The cytotoxicity of AZD5335 was affirmed *in vitro* in FRα-positive tumor cells lines (IC_50_ values 0.5-5 nM), and so was its bystander potential. In accordance with the mechanism of action of the payload drug, AZD5335-treated cells exhibited increased levels of DNA damage markers. The plasma elimination half-life of AZD5335 was calculated in humanized FcRn mice and found to be 8 days, with serum levels returning to their lower limit 21 days after administration. For the *in vivo* evaluation of AZD5335, four gynecologic cancer xenograft models were established with varying FRα expression levels. KB- and IGROV-1-based tumors, displaying ≥75% of viable tumor cells with ≥2+ FRα staining intensity, were considered as high-FRα. SKOV-3 tumors with <25% of viable tumor cells with any staining intensity, were classified as ultra-low FRα. While the intermediately stained OVCAR-3 tumors were classified as low-FRα. Remarkably, a single 5 mg/kg dose of AZD5335 achieved 100% and 95.55% TGI of KB- and IGROV-1-based tumors, respectively, inducing complete and durable regression for over 90 days. In the ultra-low FRα group, AZD5335 achieved TGI of 53.37% (*p* = 0.0003) 219. This AZD5335-dependent TGI effect in ultra-low-FRα SKOV-3-based xenografts was much higher than observed with MIRV 160, or with STRO-002 in comparable OV-90 tumors 194. Indeed, a head-to-head comparison was conducted between AZD5335 and MIRV as monotherapies in three xenograft models classified as MIRV-ineligible, i.e., <75% of viable tumor cells with ≥2+ FRα staining intensity 219. In CTG-3226- and OV0857-CIS-based xenograft models, an AZD5335 dose of 2.5 mg/kg was sufficient to induce ~ 95% TGI (*p* = 0.0004 and *p* = 0.0054), while MIRV induced a non-significant 30% and 2% TGI, respectively. In OVCAR-3-based xenograft models, the difference between the anti-tumor activity of AZD5335 and MIRV was nullified at a dose of 5 mg/kg, with both achieving ~ 68% TGI (*p* = 0.0007 and *p* = 0.0013, respectively). The authors established MIRV-resistant-OVCAR-3 tumors by continuous biweekly dosing of OVCAR-3 xenografts with 5 mg/kg MIRV. When MIRV-resistant OVCAR-3 xenograft models were re-challenged with 5 mg/kg AZD5335, 4/4 regressed following the first AZD5335 dose, and 3/4 achieved complete regression following the second dose. These results indicate that AZD5335 is more efficacious than MIRV in a wider range of FRα-expressing tumors. The *in vivo* anti-tumor activity of AZD5335 as a monotherapy was further evaluated in 17 PDX models, originating from primary and metastatic OC tumors 219. According to their FRα staining, of the 17 PDXs, 4 were considered high-FRα, 11 were classified as low-FRα, and 2 were ultra-low-FRα. A single 5 mg/kg dose of AZD5335 achieved an ORR of 76% (i.e., ≥30% reduction in tumor volume from baseline). The PDX responders, half of which displayed 100% tumor regression, included the 4 high-FRα and 9 of the low-FRα, however, the two remaining low-FRα PDXs exhibited complete tumor stasis. No apparent benefit could be concluded for the AZD5335 treatment in the 2 ultra-low-FRα PDX models 219.

FONTANA, a phase I/IIa, open-label study is ongoing to assess the safety, tolerability, and preliminary clinical activity of AZD5335 in patients with solid tumors (NCT05797168). Initial results obtained from PROC patients, without prior selection for FRα expression, who received AZD5335 as monotherapy once every three weeks at a dose range of 0.8‒3.0 mg/kg have been presented 224. While grade ≤2 nausea was a common (61%) side effect of AZD5335 treatment, grade ≥3 adverse effects were minor (18% anemia) with no dose-limiting toxicity reported. Among eight patients, with the above described high-FRα-expressing tumors, 5 exhibited an objective radiological response. In a follow up report with over 120 patients 225, dose reduction due to TEAEs occurred in 19.7% of the participants and discontinuation in 7.1%. Grade ≥3 neutropenia developed in 22.9% of the patients. ORRs of 60.7% and 47.5% were achieved in the high-FRα (n = 56) and low-FRα (n = 61) groups, respectively. Undoubtedly, these are the best response rates demonstrated thus far for low-FRα-expressing tumors with an FRα-targeted monotherapy. A remarkable clinical case study was reported on a 66-year-old patient with stage IIIC serous OC, whose tumor exhibited low FRα expression (i.e., ≥25% of viable tumor cells with ≥1+ staining intensity and <75% of viable tumor cells with ≥2+ staining intensity) 219. The patient, receiving 2.4 mg/kg AZD5335 every 3 weeks, achieved a PR after 6 weeks, and proceeded to a CR after 8 months. The superiority of ZD5335 as a monotherapy will be evaluated in a large-scale (n > 1000) clinical trial, which is currently recruiting participants for the comparison of AZD5335 with either MIRV in high-FRα PROC or NPSAs in low-FRα PROC (NCT07218809). Additional FRα-targeted ADCs which are under evaluation are reviewed by Yi Liu et al., 226.

### 3.2. FA-conjugates

#### 3.2.1. FRα-selective PROteolysis TArgeting Chimeras (PROTACs)

Proteolysis targeting chimeras (PROTACs), first pioneered and introduced by Craig Crews, Raymond Deshaies and Kathleen Sakamoto 227, are chimeric molecules that target a protein of interest (POI) for ubiquitination and degradation 228,229. Designed to treat a spectrum of diseases, PROTACs constitute a novel modality for targeted cancer therapy 230,231. PROTACs consist of two covalently linked ligands, one targeting a POI and the other engages an E3 ubiquitin ligase (E3) 232. The POI ligand selectively binds the target protein and brings along the linked E3 ligand. The latter recruits an E3 in close proximity, thereby facilitating the usage of the ubiquitin proteasome system (UPS) 233 for the selective degradation of the POI. In contrast to pharmacological enzyme inhibitors which require prolonged binding to their target in order to elicit their occupancy-dependent inhibitory activity, PROTACs have an event-based mechanism of action. Specifically, PROTACs need to bind their POI for as long as it takes for the E3 to catalyze the ubiquitination process, i.e., attach a ubiquitin to a lysine site in the targeted protein. Following steady state detachment from- or degradation of the POI, the PROTAC is free to re-engage an additional substrate. Hence, this sub-stoichiometric activity along with their selective targeting, allows PROTACs to be administrated at lower drug doses, reducing possible TEAEs and highlighting them as a promising therapeutic modality.

**Figure 2:**
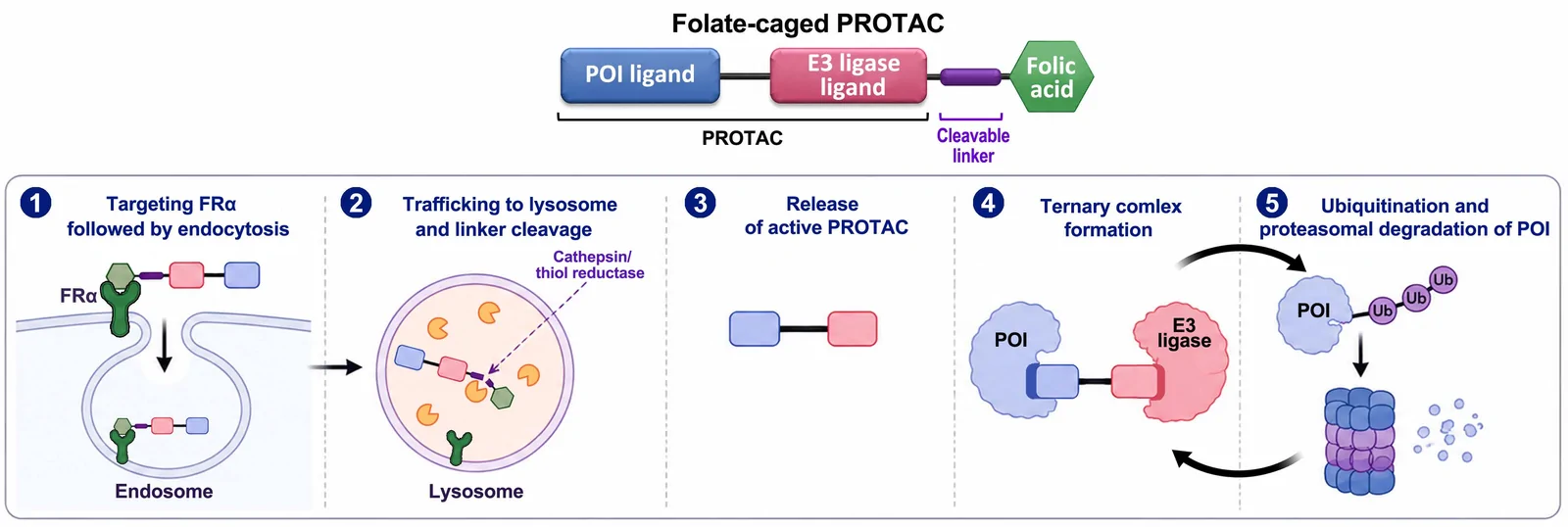
**Structure and mode of action of folate-caged PROteolysis TArgeting Chimeras (PROTACs).** The PROTAC consists of a ligand for an intracellular protein of interest (POI) that is covalently linked to an E3 ubiquitin ligase ligand. The latter is attached via a cleavable linker to folic acid (FA), that renders the PROTAC inert. 1) The folate-caged PROTAC targets cancer cells via binding to FRα on the cell surface, following which, the complex undergoes receptor-mediated endocytosis. The complex is transported from the plasma membrane via an endosome which fuses with a lysosome. 2) Under the acidic conditions within the lysosome, FA dissociates from FRα, which is then free to recycle to the plasma membrane. Depending on the specific FA linker used, the latter is cleaved by active lysosomal components to release the PROTAC; disulfide linkers are reduced by thiol reductase or glutathione, ester bonds are cleaved by esterases, and cathepsin-sensitive linkers by cathepsins. 3) The activated PROTAC exits the lysosome into the cytosol. 4) The POI ligand selectively binds the target protein and brings along the linked E3 ligand, which recruits an E3 ubiquitin ligase in close proximity, thereby forming a ternary complex. 5) The POI undergoes ubiquitination and subsequent degradation via the proteasome machinery, releasing the PROTAC to re-engage a new POI substrate.

Since many cancer POIs are intracellular and are often expressed by- and essential for healthy cells as well, a strategy was developed for the targeted internalization and activation of PROTACs selectively in cancer cells, termed caged PROTACs 234,235. The caging approach includes the addition of a conjugated appendage group to either of the ligands within the PROTAC, hence preventing their interaction with the POI/E3 and rendering the PROTAC inert, similarly to a prodrug. Unlike the short and stable linker between the two PROTAC ligands, the caging group's linker is long and cleavable by intracellular hydrolases or proteases, allowing the release and activation of the PROTAC. In the case of folate-caged PROTACs, the FA moiety plays three functions: 1) A high affinity homing molecule which targets the PROTAC to FRα-expressing cancer cells. 2) An entry key that activates RME of the PROTAC via FRα. 3) An inactivation appendage that is enzymatically removed upon hydrolysis within the lysosome. A scheme illustrating the mode of action of FRα-caged PROTACs is depicted in Figure 2.

In this context, the Wenyi Wei group developed three folate-caged PROTACs 236 based on the previously described chimeras ARV-771 237,238, MS432 239 and MS99 240. These three PROTACs are based on an E3 Von Hippel-Lindau (VHL)-binding moiety 241, linked to either a bromodomain and extra-terminal (BET) protein-binding triazolo-diazepine acetamide moiety 242, a portion of the Raf-activated MAP/ERK kinase (MEK) inhibitor PD0325901 243, or the anaplastic lymphoma kinase (ALK) inhibitor ceritinib 244, respectively. BET proteins are epigenetic readers that regulate gene transcription during various stages of cellular proliferation and differentiation 245. BET proteins are attractive targets for cancer therapy 246,247, since their inhibition was shown to modulate the suppressive TIME 248-250 as well as abolish the self-renewal ability of pluripotent stem cells and induce their differentiation 251. MEK1/2 are mitogen-activated kinases of serine, threonine and tyrosine, that are referred to as the “gatekeepers” of ERK activity 252. As such, MEK1/2 are validated therapeutic targets 253, with five FDA approved inhibitors for the treatment of cancers harboring BRAF mutations like malignant melanoma and NSCLC 254. ALK is a transmembrane tyrosine kinase receptor involved in the tumorigenesis of several human cancers 255, and hence has been the target of various FDA approved inhibitors, including for the treatment of NSCLC 256. Thus, the three POIs chosen by the Wenyi Wei group are well-deserved degradation targets as an anti-cancer strategy. Within the folate-caged PROTACs, the FA moiety was conjugated to the E3 ligand via an ester bond, thus blocking the ability of the chimeras to mark the POIs for degradation 236. Folate-ARV-771 was incubated in cell culture medium supplemented with 10% FBS for 16 h to evaluate its stability. Spontaneous and esterase-mediated hydrolysis released 20% of the PROTAC within this incubation period, suggesting a different cleavable linker should be used to reduce off-target toxicity. The ability of ARV-771 to induce intracellular degradation of the BET proteins bromodomain-containing protein 3 (BRD3) and BRD4 has already been verified in human prostate carcinoma cells with a remarkable DC_50_ (drug concentration to induce 50% protein degradation) of < 1 nM 237. Hence, a head-to-head comparison between ARV-771 and its folate-caged counterpart was performed, using high-FRα-expressing cancer cells and non-malignant FRα-null cells 236. In FRα positive HeLa, OVCAR-8 and T47D cells both BRD degraders exhibited comparable DC_50_ values, with a slight advantage for the free ARV-771. Complete BRDs degradation was observed after 1-2 h. In the FRα-null 3T3 and HK2 cells, folate-ARV-771 exhibited a minor effect on BRDs degradation, whereas in human HFF-1 fibroblasts the DC_50_ of folate-ARV-771 was only 10-fold higher than that of ARV-771 236. ARV-771 has also been shown to reduce the RNA level of c-Myc, resulting in decreased c-Myc protein levels and induction of caspase-dependent apoptosis 237. Thus, the cytotoxicity of folate-ARV-771 was evaluated 236. Consistent with their degradation capacity, ARV-771 and folate-ARV-771 exhibited comparable IC_50_ values in the FRα-expressing cells, with the lowest IC_50_ values observed in the highest FRα expressing cell line T47D (i.e., 13 nM vs. 18 nM, respectively). In contrast, in the FRα-null cells, ARV-771 was 6-12-fold more efficient than folate-ARV-771. The cytotoxicity elicited by the folate-caged degrader in these FRα-null cells could be the result of both the instability of the ester bond and cell targeting via FRβ. Interestingly, a cleavage-resistant analogue of folate-ARV-771 had neither degradation activity nor cytotoxicity, establishing the instability of the ester bond as the culprit, as well as validating the blocking capability of the FA moiety. Similar results were obtained with the other folate-caged PROTACs, and their targeted POIs 236. Folate-MS99 elicited the degradation of transforming ALK-fusion proteins commonly found in cancer. These included EML4-ALK in NSCLC 257 and NPM-ALK in lymphoma 258, both of which render ALK constitutively active and can confer resistance to ALK tyrosine kinase inhibitors (TKIs) 259,260. Folate-MS432 efficiently elicited the degradation of MEK1 and MEK2 236. Both caged-PROTACs degraded their target proteins and elicited cell cytotoxicity in an FRα- and proteasome-dependent manner. FA-S2-MS4048, an additional ALK-targeted folate-caged PROTAC has been synthesized and evaluated 261.

With an intention to enhance solubility, biocompatibility, stability and circulation time for *in vivo* experiments, the concept of caged PROTACs evolved into PEGylated PROTAC nanoparticles (NPs) 262-264, where the core chimeras are coated with polyethylene glycol (PEG) 265. In this respect, self-assembling micelles of FA-PEG-PROTAC (MPRO) were designed for the reduction-responsive proteolysis of EGF receptor (EGFR) 264. MPRO consists of the previously published VHL E3-based PROTAC MS39, which utilized the TKI gefitinib 266 for selective targeting and degradation of mutant EGFR in lung cancer cells 267. Activating somatic mutations in the tyrosine kinase domain of the EGFR gene are frequent in NSCLC patients 268, mostly in exon 19 269. The E3 ligand, within MS39, was conjugated via a glutathione (GSH) cleavable disulfide linker to the commercially available FA-PEG_3400_, to generate an inert FA-caged PROTAC, which self-assembled into micelles following solvent evaporation 264. As before, an MPRO-C negative control was also synthesized harboring a non-cleavable alkyl linker; however, the hydroxyl part of the VHL E3 ligand harbors an ester bond which could reduce the stability of the linker. MPROs displayed relatively uniform spherical structures with diameters of ≈ 50 nm, and a zeta potential of -7.1, which should contribute to stability and prevent aggregation. Indeed, MPROs retained their size during a 48 h incubation in 10% FBS and up to 10 days incubation in PBS. By utilizing MPROs loaded with a fluorescent dye, the authors demonstrated the FRα-dependent cellular internalization of the micelles 264. MPRO treatment of the FRα expressing human NSCLC cells PC-9 and HCC827, which harbor the exon 19 delE746-A750 EGFR mutation 270,271, resulted in a dose-dependent degradation of EGFR 264. MS39 was superior at inducing EGFR degradation compared to MPRO, and MPRO-C was the least efficient. The higher-than-expected activity of MPRO-C was probably due to the hydrolysis of its ester bond. The relative activity of the three compounds was consistently demonstrated in *in vitro* cytotoxicity, with MPRO exhibiting no cytotoxic effect in FRα-null cells. For *in vivo* evaluation, HCC827 tumor-bearing mice were administered every other day, for 16 days, with either MPRO, MPRO-C or MS39 at a dose equivalent to 15 mg/kg MS39. While MPRO and MPRO-C were dissolved in saline, the non-soluble MS39 required a solution containing DMSO, ethanol and Tween 80, demonstrating the contribution of the PEGylated micelles to the solubility and biocompatibility of the PROTAC. MS39 and MPRO-C induced 48.1% and 58.2% TGI, respectively, while MPRO achieved 79.6% TGI, accompanied by significant reduction in EGFR expression 264.

#### 3.2.2. FRα-selective LYsosome TArgeting Chimeras (LYTACs)

**Figure 3:**
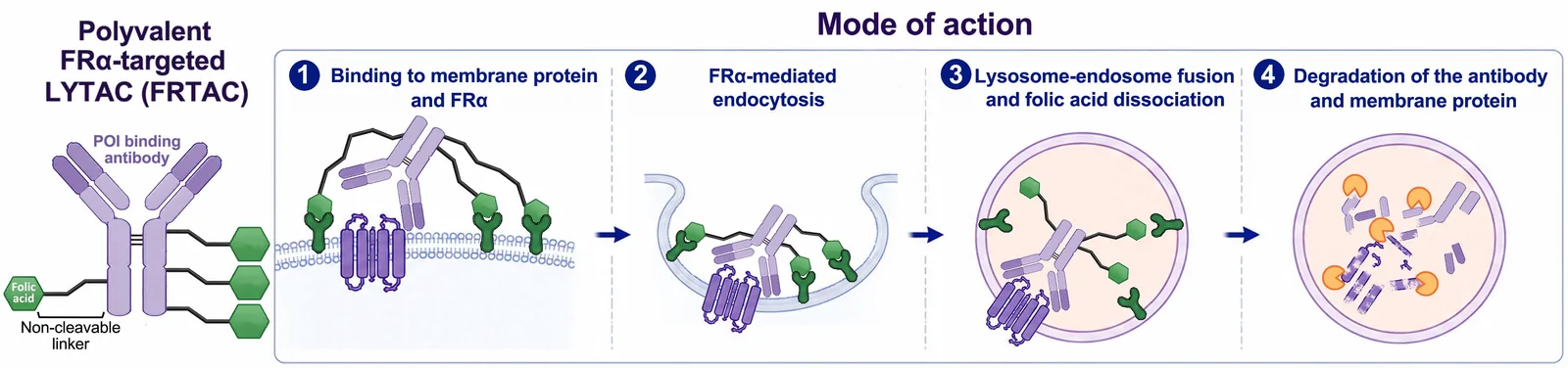
** Structure and mode of action of polyvalent FRα-targeted LYsosome TArgeting Chimeras (LYTACs) -FRTACs.** The FRTAC consists of a mAb against an extracellular protein of interest (POI), covalently linked to 4-8 folic acid (FA) moieties. 1) The FRTAC targets cancer cells via binding to several FA receptors on the cell surface, while the mAb binds to the membrane POI. 2) Following binding, the complex undergoes FRα-mediated-endocytosis and is transported from the plasma membrane via an endosome. 3) Following fusion with a lysosome, FA dissociates from FRα under the acidic lysosomal pH, and FRα recycles to the plasma membrane. 4) The mAb and POI are degraded by lysosomal cathepsins. The same mechanism also applies to soluble extracellular POIs.

An additional group of proteolysis targeting chimeras are the LYTACs which utilized the hydrolytic machinery of lysosomes for the degradation of the POI, instead of the proteasome 272. First introduced in 2020 273, LYTACs were designed for the targeted proteolysis of extracellular- and membrane proteins via endocytosis and consequent delivery into lysosomes 274. The frequent increased acidification of lysosomes in cancer cells, as well as those of tumor associated immune cells, 147,275, along with the elevated levels of proteases and hydrolase activities therein, render these cells ideal targets for LYTACs. The latter are conjugates composed of a ligand for a lysosome-shuttling membrane receptor and an antibody (or ligand) against the extracellular domain of a POI 276. The tethering of the two proteins by the LYTAC results in their co-engulfment and endocytosis, followed by their fusion-based transport into lysosomes. The low luminal pH within the lysosome induces the dissociation between the receptor and its ligand, with the former recycling back to the cell membrane ready to engage another LYTAC. In the context of the present review, FRα was exploited for the selective targeting of FRα-overexpressing cancer cells by FA-containing LYTACs, while constituting their lysosome-shuttling membrane receptor 277-279. A scheme illustrating the mode of action of FRα-targeted LYTACs is depicted in Figure 3.

Following proof of concept experiments, establishing the ability of FA-containing LYTACs (i.e., Folate Receptor TArgeting Chimeras, FRTACs) to mediate the FRα-, FRβ- and lysosomal-dependent degradation of soluble extracellular proteins, Yaxian Zhou *et al.,* synthesized cetuximab-containing FRTACs (Ctx-FA) 277. Cetuximab (Ctx, Erbitux) is a recombinant chimeric mAb against EGFR, used in the treatment of squamous cell carcinoma of the head and neck and metastatic colorectal cancer 280,281. Since all experiments in this paper 277 were performed in full growth medium, caution should be taken regarding the ability of the described FRTACs to compete with free FA for binding to FRs. In this respect, DMEM and RPMI-1640 growth media contain ~ 9 µM and 2.3 µM FA, respectively, hence far exceeding the 10-100 nM concentrations of FRTACs used in the *in vitro* experiments. Assessment of EGFR degradation following treatment of FRα- and FRβ-expressing squamous cell carcinoma and cervical cancer Fadu and HeLa cells with Ctx-FA revealed a dose- and time-dependent degradation of EGFR, with increased activity associated with higher degree of folate labeling 277. Ctx linked by a 2k-PEG-linker to the highest number of ligands, i.e., 4-5, elicited the highest degree of EGFR degradation. In addition, EGFR degradation efficiency was correlated with the FR:EGFR expression ratio. Significant degradation of EGFR was noticeable after ~ 6 h, and the maximal ~ 80% degradation activity (D_max_) of Ctx-FA remained stable for at least 72 h, with DC_50_ values of 0.41 and 0.24 nM in Fadu and HeLa cells, respectively. Importantly, fluorescence microscopy clearly showed that following treatment with Ctx-FA, EGFR from the plasma membrane of Fadu cells translocated to the lysosomes. While Ctx-FA induced the degradation of EGFR, the levels of FRα and FRβ were not affected, indicating the lysosomal release of the receptors and consequent recycling to the plasma membrane. Two additional FRTACs were synthesized, Atz-FA and Ab2-FA for the targeted degradation of PD-L1 and CD47, respectively. Atz-FA harbors the PD-L1 targeting mAb atezolizumab (Atz, ecentriq), which is the first ICI approved for the treatment of TNBC 282, and has been approved as therapy for various cancers, including alveolar soft part sarcoma 283, hepatocellular carcinoma 284, urothelial carcinoma 285 and NSCLC 286. Ab2-FA harbors a commercially available mouse anti-CD47 mAb. Treatment of TNBC and NSCLC cell lines MDA-MB-231 and A549, respectively, with either of the FRTACs resulted in the degradation of their cognate POI 277. The degradation activity of Ctx-FA and Atz-FA was compared between FR-null human keratinocyte HACAT cells and FR-expressing hepatocellular carcinoma HuH7 cells, which express comparable levels of EGFR and PD-L1. The FRTACs induced negligible POI degradation in HACAT cells while dramatically degrading their POI in HuH7 cells, establishing FRTACs as a platform for the FR-dependent degradation of proteins harboring extracellular domains. For the purpose of *in vivo* evaluation, a mouse homologue of Atz-FA was synthesized (i.e., Ab3-FA) using a commercially available rat anti-mouse PD-L1 mAb instead of Atz 277. This was puzzling since Atz is known to recognize murine PD-L1 with high affinity 287-289.

Although ICIs transformed therapy of various cancers, they are not without limitations. The expression of PD-L1 on healthy cells renders them targets of PD-L1 antibodies as well, thus PD-1/PD-L1 blockade is associated with multiple TEAEs, most of which are immune-related adverse events 290 and the onset of endocrine dysfunction 291. Particularly, PD-L1 inhibitors can even provoke a rapid onset of hypothyroidism and type 1 diabetes 292. Thus, the FR-selective PD-L1-targeting achieved by a FRTAC should alleviate this untoward toxicity. Following the validation of murine PD-L1 degradation by Ab3-FA in murine colon carcinoma and melanoma CT26 and B16F10 cells, respectively, as well as the PD-L1 translocation into lysosomes, initial *in vivo* pharmacokinetics were measured 277. Ab3-FA harboring 4-5 FA moieties per antibody, which displayed the highest degradation efficiency in a cell-based assay, also exhibited the shortest circulation half-life in C57BL/6 mice, i.e., 15 h, and thus was chosen for further experiments in tumor bearing mice. Since Ab3-FA was completely cleared from the plasma within 48 h, the treatment regimen included the administration of 2.5-7.5 mg/kg free Ab3 or Ab3-FA every other day for a total of 5 doses. The *in vivo* testing was conducted in syngeneic mouse models, harboring established tumors (~ 100 mm^3^) from three cancer origins, i.e., the previously tested CT26 and B16F10 cells as well as the oral squamous cell carcinoma MOC1 cells. In all three mouse models, Ab3-FA induced a TGI which was superior to the free Ab3 (*p* < 0.05), with complete MOC1-based tumor stasis till the end of the experiment (day 30). Analysis of resected treated tumors confirmed the near-complete degradation of tumor PD-L1 by Ab3-FA, in addition to a dramatic increase in tumor infiltrating CTLs. These results established the enhanced anti-tumor effect of PD-L1 degradation compared to PD-L1 blockade. Spleen and lung tissue biopsies from treated mice were examined via IHC and Western blot assays, where the non-malignant cells exhibited neither morphological changes nor reduction in PD-L1 expression levels; further establishing the FR-selective activity of the Ab3-FA FRTAC 277.

A second research group synthesized FRTACs with a higher number of FA moieties per antibody, termed polyvalent FRTACs 278. As before, EGFR and PD-L1 were targeted via the Ctx and Atz mAbs, respectively, to produce the FRTACs FR-Ctx and FR-Atz. Additionally, the human epidermal growth factor receptor 2 (HER2) was targeted via the trastuzumab (Ttz, Herceptin) mAb, which constitutes the standard first-line treatment for HER2-positive advanced breast cancer 293, to generate FR-Ttz 278. Lastly, trophoblast cell surface antigen-2 TROP2 was targeted via the sacituzumab (Stz) mAb 294, used in the first-in-class TROP-2 targeted ADC sacituzumab govitecan, approved for the treatment of TNBC 295, to generate FR-Stz 278. Polyvalent FRTACs with 15-20 FA conjugates per antibody induced greater degradation of the POIs at lower concentrations than their cognate FRTACs harboring two FA molecules. This is possibly due to their greater functional affinity towards FRα 296, wrongly presented in the paper as affinity 278. While FA had a calculated *Kd* of 1.06 nM to FRα, which is in agreement with previous publications discussed in the introduction, the dissociation constant decreased to 0.15 nM for FR-Ctx harboring two FA moieties, and was further reduced to 0.018 pM with the conjugation of 15 FA molecules. These values demonstrate the combined effect of increased avidity and residence time achieved by polyvalent ligands 296 and erroneously misrepresented as mere “affinity” values. It should be noted that multivalent decoration of an antibody with multiple FA residues will necessarily increase the cumulative binding capacity of the conjugate without altering the mere singular interaction of each FA residue with FRα that in fact denotes its genuine *Kd* value. The increased functional affinity of FR-Ctx, harboring 15 FA moieties, towards FRα decreased its EGFR DC_50_ value (0.099 nM) by 2-4-fold compared to that of Ctx-FA, harboring 4-5 FA molecules, (0.24-0.41 nM), resulting in the same D_max_ ~ 80% 278. FR-Atz, FR-Stz and FR-Ttz at a FA:antibody ratio of 15 exhibited DC_50_ values of 0.08, 0.93 and 1.18 nM towards their cognate POIs, respectively. All FRTACs displayed FRα-dependent activity and, as before, had no effect on FRα protein levels.

Unless otherwise stated, the following text refers to FRTACs with a FA:antibody ratio of 15. The human lung mucoepidermoid carcinoma H292 cells (FRα^+^ EGFR^+^ PD-L1^+^) were used to evaluate the downstream effects of the FRTACs compared to their free mAb counterparts. FR-Ctx blocked the proliferative impact of EGF, as well as its downstream kinase signaling axis, resulting in minute levels of phosphorylated STAT3 and ERK1/2, in a manner exceeding free Ctx. Co-culturing H292 cells with activated human-derived PBMCs under treatment with FR-Atz, demonstrated the ability of FR-Atz to negate immunosuppression. The FR-Atz-dependent degradation of PD-L1 allowed for the dramatic activation of CTLs, resulting in an increased secretion of granzyme B and interferon-gamma (IFNγ), leading to tumor cell apoptosis. Free Atz had little effect on PD-L1-dependent immunosuppression. Using H292-tumor bearing mice and whole-animal body imaging, fluorescently tagged FR-Atz was shown to accumulate at the tumor site within 5 h, and retained fluorescence intensity for 5 days. In contrast, free Atz had a slow tumor accumulation rate of 1-2 days. Tumor FR-Atz fluorescence intensity far exceeded that of any healthy organ, and consistently, FR-Atz-dependent degradation of PD-L1 was achieved solely in the tumor, indicating an FRα-selective mode of action. The FR-Atz-dependent degradation of tumor PD-L1 was also demonstrated in mouse prostate cancer RM-1 cells (FRα^+^ PD-L1^+^), both *in vitro* and *in vivo*. In RM-1-tumor bearing mice, degradation of PD-L1 following FR-Atz treatment resulted in apoptosis of tumor cells and a consequent ~ 75% reduction in tumor weight (*p* = 0.0003). Lastly, using mouse models harboring tumors established from B16F10 melanoma cells expressing human PD-L1, the authors confirmed the FR-Atz-dependent reprograming of the immunosuppressive TIME. This included polarization of tumor associated macrophages (TAMs) from the M2 to the M1 phenotype, activation and tumor infiltration of CTLs, as well as reduction in the levels of regulatory T cells 278.

Following the promising results obtained with its published PROTACs, the Wenyi Wei group recently presented a novel design of an FRTAC developed to hijack FRα for the simultaneous degradation of a pair of complementary transmembrane POIs, i.e., EGFR/HER2 or PD-L1/VISTA 279. EGFR and HER2, both members of the ErbB family of growth factor receptors 297, can form highly active heterodimers which instigate tumor cell proliferation 298-300. A frequent mechanism of resistance to Ttz is EGFR overexpression 301-303, while HER2 hyper-activation was shown as a mechanism of Ctx resistance 304,305. Hence, the simultaneous targeting of both receptors can enhance the therapeutic efficacy. In this respect, dual inhibitors have been approved for clinical use, such as the irreversible ErbB family blocker afatinib 306. However, HER2 and EGFR are expressed in a wide variety of healthy tissues 109,307, therefore, inhibiting their signaling pathways can result in TEAEs. Indeed, like other ErbB-directed mAbs, Ctx and Ttz inflict various severe TEAEs including dermatologic toxicities and interstitial pneumonitis 308. In fact, 70% of colorectal cancer patients treated with Ctx developed severe TEAEs 309. The newly designed FRTAC that targets the EGFR/HER2 duo, i.e., FolTAC-dual, should overcome both problems of acquired resistance and off-target toxicity 279. The same is true for the pair of V-domain immunoglobulin suppressor of T-cell activation (VISTA) 310 and PD-L1, both of which are negative immune checkpoint molecules, that are expressed in healthy tissues 311. Within the TME, both tumor and immune cells frequently express the VISTA/PD-L1 duo, resulting in enhanced immune resistance 311. Although they represent distinct immune checkpoint pathways, they can compensate for one another. Thus, following anti-PD-L1 treatment, disease progression in melanoma patients is associated with increased density of VISTA+ lymphocytes (*p* = 0.009) 312. Consistently, bispecific antibodies designed on the basis of Atz and onvatilimab (CI-8993), an anti-VISTA mAb currently evaluated in clinical trials 313, for the co-inhibition of PD-L1 and VISTA, exhibited superior anti-tumor activity than either monotherapy or combination therapy 314. The Wenyi Wei group first constructed single degrader FolTACs for each POI, harboring a farletuzumab scFv linked to either a HER2 binding affibody ligand 315, EGFR affibody ligand 316, Atz scFv, or onvatilimab scFv, with remarkable D_max_ = 82-95% 279. Then, for the assembly of the FolTAC-duals, two geometries were explored. The “knob-into-hole” geometry consisted of the two POI targeting moieties on separate sides of the construct, while in the “string” geometry both sides of the construct harbored the two targeting moieties connected one after the other. The “string” configuration was found to enhance both the binding affinity to the POIs and the degradation efficacy in both FolTAC-duals, which successfully induced the FRα- and lysosome-dependent co-degradation of their POI pairs *in vitro* 279.

Next, the ability of the FolTAC-duals to surmount drug resistance elicited by inhibition of their POIs was evaluated. To this end, Ttz-resistant human breast cancer SK-BR-3-pool2 cells 317 were further exposed to 0.25 µM lapatinib (Lap) for 8 weeks to establish Ttz/Lap cross-resistant cells (FRα^+^ EGFP^+^ HER2^+^) 279. Lap is a small molecule TKI that blocks HER2 signaling by competitively binding the intracellular ATP-binding sites of HER-2 318. The EGFR/HER2 FolTAC-dual induced high apoptosis rates in the resistant cells, comparable to that in the sensitive parental cells, while normal human foreskin HFF-1 fibroblast cells were not affected 279. *In vivo* experiments conducted in mouse models bearing Ttz/Lap resistant SK-BR-3-based tumors, revealed the enhanced anti-tumor activity of the FolTAC-dual compared to an anti-HER2 FolTAC. FolTAC-dual induced complete tumor stasis, while HER2-FolTAC induced a non-significant TGI. It should be conveyed that the combination of HER2-FolTAC and EGFR-FolTAC had a dramatic anti-tumor effect as well. The PD-L1/VISTA FolTAC-dual was evaluated using an MC38 syngeneic mouse model, where the MC38 cells were engineered to express human FRα and VISTA. PD-L1/VISTA FolTAC-dual induced complete degradation of both POIs only in 3/7 tumors evaluated by Western blot and this correlated with the low survival rates of the tested mouse models 3/15, although half exhibited TGI and ~ 2-fold longer survival time 279. FRα-guided proteolysis targeting chimeras show great potential as a selective and efficient therapy mode for FRα-positive tumors, and should be further evaluated pre-clinically to establish feasible clinical value.

## 4. FRβ-dependent reprogramming of the suppressive TIME

As mentioned above, and detailed in our recent review 147, the predominant tumor-associated immune cell population of macrophages 319,320 is of the M2 phenotype that orchestrates cancer immune evasion and immunosuppression 321-323. M0 monocytes can undergo polarization by naturally occurring signaling cues which have been exploited for immune research 324. Specifically, macrophages assume the M1 configuration following stimulation by microbial agonists of toll-like receptors (TLRs), e.g., lipopolysaccharide (LPS), as well as pro-inflammatory cytokines such as IFNγ and TNF; whereas polarization to M2 macrophages is promoted by IL-4 and TGF-β. Unfortunately, the tumor associated population of M2 macrophages also increases following anti-cancer treatments, including radiotherapy 325,326 and chemotherapy based on cisplatin 327-329 and cyclophosphamide 330. Although some ambiguity exists regarding macrophage polarization following activation with the cytokine granulocyte-macrophage colony-stimulating factor (GM-CSF) and macrophage colony stimulating factor (M-CSF), it is largely accepted that GM-CSF promotes the transition of macrophages to the M1 phenotype, whereas M-CSF favors M2 331-333. It has been established that by attenuating the activity of lysosomes within monocytes and macrophages, one can polarize these cells to the M1 configuration and induce an anti-tumor immune response 147. Lysosome dysfunction can be achieved, for instance, by alkalization and lysosome membrane permeabilization (LMP) 147,334-336.

*In vitro* experiments, where human-derived monocytes were polarized to either the M1 or M2 phenotype, by the various factors detailed above, allowed for the characterization of “pure” macrophage populations 26. One dramatic variable found was the differential expression of FRβ on M2 macrophages, enabling them to bind and internalize FA. Moreover, TAMs within patient-derived melanoma specimens were positive for cell surface FRβ, and so were stromal cells of lung, ovary, colon, gastric, and breast cancers 26. Hence, the expression of FRβ on TAMs has been further explored and studied 27, revealing that active FRβ is not just a biomarker for suppressive TAMs but also necessary for their activity 337. While FRα-targeted therapies are restricted to a small subset of tissues, the expression of FRβ on TAMs and tumor resident myeloid-derived suppressor cells (MDSC) 28 highlights it as a potential therapeutic target affecting any type of tumor. FRβ can be used for the detection of solid tumors, for targeted cytotoxicity, thereby reducing the population of immune suppressive cells, or for the targeted conversion of M2 macrophages to the M1 phenotype, which will further reprogram the TIME.

### 4.1. FRβ-targeted conversion of M2 macrophages

**Figure 4:**
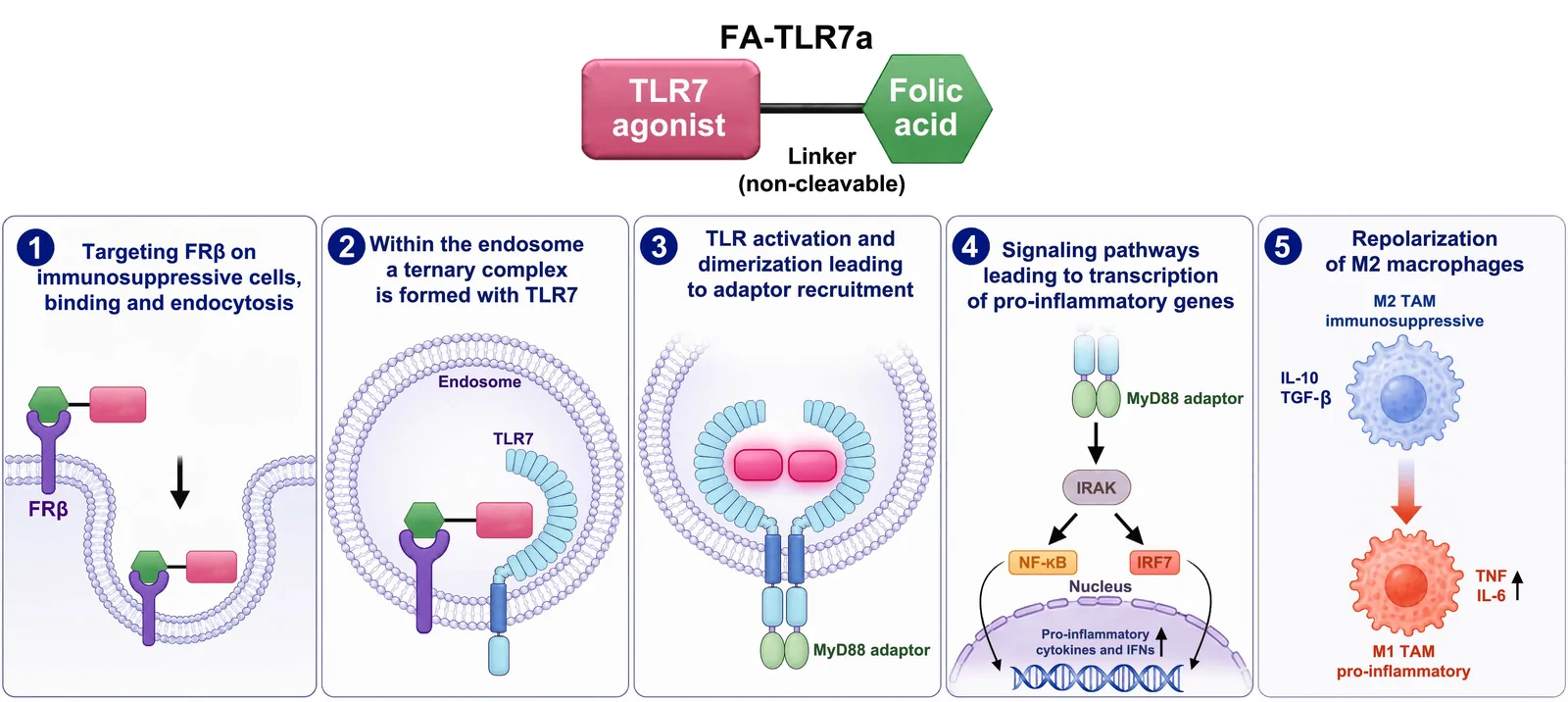
** Structure and mode of action of FA-TLR7a.** FA-TLR7a consists of a folic acid (FA) moiety covalently attached to a toll-like receptor 7 (TLR7) agonist (TLR7a). 1) FA-TLR7a binds to FRβ, expressed on the surface of immunosuppressive immune cells, primarily M2 tumor-associated macrophages (TAMs), following which the complex undergoes receptor-mediated endocytosis. 2) Within the endosome, the FRβ-bound FA-TLR7a binds the resident TLR7 to form a ternary complex. 3) Following binding to its agonist, TLR7 is activated and undergoes dimerization, resulting in the recruitment of its adaptor protein myeloid differentiation primary response 88 (MyD88), a key adaptor for innate immune response signaling. 4) MyD88 activates downstream signaling pathways resulting in the nuclear translocation of the transcription factors NF-κB and IRF7 and consequent transcription of pro-inflammatory cytokines and interferons. 5) The pro-inflammatory cues drive the repolarization of M2 TAMs to the M1 phenotype, resulting in secretion of anti-tumor cytokines IL-6 and TNF.

In an extensive study on the expression of FRβ in various tumor associated cells, using multiple diverse mouse tumor grafts, FRβ was found to be co-expressed with known markers of MDSCs and TAMs in 11-26% of total tumor cells 28. This indicated the high prevalence of tumor infiltrating suppressive cells. Mice harboring syngeneic FR^-^ MB49 bladder cancer tumors were treated with a folate-targeted photodynamic therapy (PDT) agent to eradicate FRβ-positive myeloid suppressive cells. However, full replenishment of the FRβ-positive cell population was observed within 72 h 28. This demonstrated that using FRβ for targeted cytotoxicity of immunosuppressive cells is futile, hence reprograming these cells could be a better solution. To this end, orthotopic FR^-^ 4T1 mammary tumor bearing mouse models were treated with a FA-conjugated TLR7 agonist (FA-TLR7a) (patent US20210170035A1) 28. The intracellular TLR7, expressed primarily by macrophages and DCs 338, is involved in the detection of pathogen-associated molecular patterns and induction of a host immune response. Following ligand binding by TLR7, there is an induction of conformational changes and adaptor recruitment, resulting in the nuclear translocation of the central transcription factor nuclear factor kappa-light-chain-enhancer of activated B cells (NF-κB). The latter induces the expression and secretion of pro-inflammatory cytokines, such as the M1-macrophage associated IL-6 and TNF 339. Both FA-TLR7a and free TLR7a had no cytotoxic effect on FR^-^ 4T1 tumor cells *in vitro* 28. However, the FRβ-mediated uptake of FA-TLR7a by TAMs and MDSCs *in vivo* resulted in significant TGI in the mouse models with no associated loss of mouse body weight. In contrast, free TLR7a had no effect on tumor growth but was highly toxic, as reflected by the drastic loss of body weight and systemic release of cytokines 28. Indeed, TLR7 agonists have never received approval by the FDA since they activate immune cells in healthy tissues, leading to systemic toxicity and immune complications such as neuroinflammation 340, lymphopenia 341 and cytokine release syndrome 342. The FRβ-selective targeting of TLR7 prevents a systemic reaction by confining the immune response to FRβ^+^ TLR7^+^ immune cells at the primary tumor site and any metastases. In this respect, FA-TLR7a was shown to completely circumvent the formation of metastases for 18 days (*p* ≤ 0.05), whereas free TLR7a had no such inhibitory effect 28. To validate that this FA-TLR7a-induced anti-tumor effect was achieved by reprograming of immune cells to a tumoricidal M1-like phenotype, the authors isolated TAMs and MDSCs from the treated tumor-bearing mice. Analyses showed that the expression of suppressive markers by TAMs and MDSCs was reduced by >50%, accompanied by a significant shift in macrophages from an M2 towards the M1 phenotype. A scheme illustrating the repolarization of M2 TAMs by FA-TLR7a is depicted in Figure 4.

The results further revealed the consequent activation of an immune response by a ~ 10-fold increase in the number of tumor infiltrating T helper cells and CTLs (*p* ≤ 0.001). Finally, FA-TLR7a increased the median survival time of orthotopic 4T1 tumor-bearing mice from 27 day to 37 days (*p* ≤ 0.01) 28. An additional FA-linked TLR7 agonist (FA-TLR7-1A) was designed and tested 343. The TLR7 agonist chosen was cell-impermeable and linked to FA by a hydrophilic non-cleavable PEG spacer to prevent circulation and cell entry of free agonist. FA-TLR7-1A exhibited comparable affinities of ∼ 20 nM for both TLR7 and FRβ. The activity of FA-TLR7-1A was validated *in vitro* via its ability to induce the production of TNF and IL-6 by immunosuppressive M2-like macrophages, with maximal activity occurring at 10 nM. Its pharmacological safety was assessed by monitoring the weight of treated mice during five days of FA-TLR7-1A administration (60 nmol). Control and FA-TLR7-1A-treated mice gained 5% of body weight, whereas free TLR7-1A induced a drastic 10% loss of body weight 343. These results established a therapeutic window where FA-TLR7-1A is both active and pharmacologically safe. As before, orthotopic 4T1 tumor bearing mouse models were used for *in vivo* evaluation, and were injected with 3 nmol/day. FA-TLR7-1A induced a ~ 50% reduction in the growth of the primary tumor (*p* < 0.001) and almost completely prevented the development of lung metastases (*p* < 0.01), with no effect on body weight for 20 days. Post treatment tumors were dissected and analyzed for pro-inflammatory and anti-inflammatory markers expressed by tumor infiltrating TAMs and MDSCs. Post-treatment immune cells exhibited a robust transition from an immunosuppressive state to an active state, accompanied by the activation of CTLs 343. Since it appeared that FA-TLR7-1A was not potent enough as monotherapy, the authors explored its possible combination with ICI mAbs against murine PD-1 (clone: 29F.1A12) or cytotoxic T lymphocyte-associated antigen-4 (CTLA-4, clone: 9H10) 344. T helper lymphocytes expressing high levels of CTLA-4 are reprogrammed into regulatory T lymphocytes exhibiting immunosuppressive characteristics. Anti-CTLA-4 alone induced 30% TGI in 4T1 tumor bearing mice, while the combination with FA-TLR7-1A resulted in a dramatic 90% TGI (*p* < 0.0001) 343. This efficacious combination therapy reprogrammed the suppressive TIME by converting the TAMs and MDSCs to a tumoricidal phenotype, inducing an increase in tumor infiltrating activated T helper cells and CTLs. Consistently, in syngeneic MC38 colon cancer models, the combination of FA-TLR7-1A with the anti-PD-1 mAb resulted in CR. The potent immediate activation of the reprogrammed TIME also induced prolonged immunity which protected the mice upon re-challenging with MC38 tumor cells; hence preventing the establishment of new tumors 343. A detailed demonstration of TIME reprogramming by FA-TLR7-1A has been published 345.

## 5. Co-targeting tumor and immune cells

Metabolic reprograming, now considered as the eighth hallmark of cancer 346, occurs in tumor cells in addition to the TME and the TIME, where it contributes to tumorigenesis, rapid cell proliferation, invasion, immune evasion, immunosuppression as well as drug resistance 347-353. In this respect, the metabolism of the essential amino acid glutamine (Gln) is frequently enhanced in cancer 354,355, since it serves as a critical carbon and nitrogen donor for various biosynthetic processes such as lipogenesis, *de novo* nucleotide biosynthesis and amino acid synthesis. In addition, glutaminolysis in mitochondria allows the anaplerotic refueling of the TCA cycle via provision of alpha ketoglutarate (αKG), and αKG conversion to citrate allows adaptation to hypoxia 356. On the other hand, Gln is also required for the proper activity of the immune system 353,355,357; specifically, T cells rely on Gln for rapid proliferation and immune response 355,358, and Gln regulates macrophage polarization 355,359,360. Hence selective targeted inhibition of Gln metabolism may hamper cancer cell proliferation while sparing healthy tissues and T cell populations 361.

One strategy for selective targeting of Gln metabolism utilized poly D,L-lactide-co-glycolic acid (PLGA)-PEG-FA NPs, loaded with the first-generation Gln antimetabolite 6-diazo-5-oxo-l-norleucine (DON) 362 and pH-responsive calcium carbonate (CaCO_3_) to generate (FA-DCNPs) 363. DON is a two-step inhibitor of multiple glutamine-utilizing enzymes, such as glutaminase and various glutamine amidotransferases 364. Following its competitive binding to the Gln binding active site, it forms a covalent adduct that irreversibly inhibits the enzyme. While clinical studies with DON, conducted in the 50's, were abandoned due to dose-limiting toxicity, renewed interest in the drug has arisen following the discovery of Gln-dependence in cancer 362,364. The pH-responsive decomposition of CaCO_3_ was shown to facilitate drug release from PLGA NPs under acidic conditions (pH 5.5) 365, suggesting good drug release within lysosomes. Moreover, within the acidic lysosome, the carbonate ions interact with protons leading to lysosome alkalinization and calcium (Ca^2+^) release, both of which contribute to lysosome dysfunction and LMP 336,366. As detailed in our recent review 147, M2 macrophages are characterized by highly acidic lysosomes with enhanced hydrolase activity, which promote augmented autophagic flux. M2 macrophages can be converted to the M1 phenotype by alkalinizing their lysosomes. In addition, tumor cells are also frequently characterized by acidic lysosomes and enhanced autophagic flux, resulting in over-processed tumor antigens which cannot be presented by MHC for the recognition and activation of immune cells 147. Hence, by selectively binding to both FRα-positive tumor cells and their associated FRβ-positive suppressive immune cells, FA-DCNPs have the potential to disrupt the lysosomes of tumor and immune cells, inhibit tumor cell proliferation and increase immune reactivity. The size of the FA-DCNPs remained relatively stable during a 20 days incubation in cell culture medium supplemented with 10% FBS 363. FA-DCNPs exhibited a time- and pH-dependent release of DON and Ca^2+^. Murine epithelial OC ID8 cells were verified for high expression of both FRα and FRβ, while both receptors were nearly undetectable in human umbilical vein HUVEC cells. As already established, M2 macrophages displayed high levels of FRβ, M0 monocytes exhibited moderate expression levels, while M1 cells expressed low levels, with all three subtypes displaying low levels of FRα. Accordingly, ID8 and M2 cells displayed high FA-DCNPs internalization rates (i.e., 96% and 88%, respectively) compared to the other cell types (*p* < 0.001). Specifically, the internalization rate by M1 macrophages was lower by >70% compared to M2, whereas HUVEC cells did not exhibit any internalization. *In vitro* cytotoxicity assays established the FR-dependent killing mechanism of FA-DCNPs, which included a 6.4-fold increase in cellular reactive oxygen species (ROS) levels 363. Perturbation of redox homeostasis is an established consequence of disrupted Gln metabolism, following the reduction in cellular anabolism, as well as glutamate and GSH levels 356,367. Apart from the disruption of Gln metabolism by DON, FA-DCNPs also elicited cytosolic release of Ca^2+^ which triggered mitochondrial Ca^2+^ overload and consequent dysfunction 363, thus further contributing to the oxidative stress 368. It is surprising that the authors completely disregarded the contribution of the apparent FA-DCNPs-dependent LMP that facilitated the release of Ca^2+^ and ROS from lysosomes, hence triggering mitochondrial Ca^2+^ overload. Since mitochondrial damage was shown to induce ICD 369-372, the authors explored whether this is also relevant to FA-DCNPs. Indeed, following treatment with FA-DCNPs, ID8 cells exhibited high levels of the canonical ICD-associated DAMPs, including >10-fold increase in surface expression of CRT and a ~ 3-fold increase in HMGB1 release (*p* < 0.001) 363. Given that macrophage M1 polarization can be induced by lysosomal dysfunction 147, oxidative stress-damaged mitochondria 372, deregulated Gln metabolism 373,374 and ICD 375, the ability of FA-DCNPs to rewire M2 macrophage was evaluated. M0 RAW264.7 cells polarized to M1 and M2 populations by LPS and IL-4, respectively, were analyzed for their glutamate content 363. Following FA-DCNPs treatment, M2 macrophages exhibited a ~ 60% drop in their glutamate content and underwent robust reprograming into the M1 phenotype, presented by the co-stimulatory CD86 surface biomarker and secretion of IL-12. Importantly, treatment of RAW264.7 cells with FA-DCNPs prior to their stimulation with IL-4, skewed their polarization into M2 macrophages, consistently resulting in M1 cells.

*In vivo* evaluation of FA-DCNPs was conducted in ID8-based xenograft mouse models 363. Compared to nontargeted DCNPs, FA-DCNPs exhibited a higher tumor:liver accumulation ratio after 48 h, albeit the liver accumulation was substantial. Nonetheless, blood tests demonstrated normal liver function for 28 days, and IHC showed no aberrant cellular morphology. FA-DCNPs, administered every other day for a total of 4 doses, induced a dramatic inhibition of tumor cell proliferation along with enhanced tumor cell apoptosis (*p* < 0.001). During the treatment, FA-DCNPs markedly prevented tumor growth (TGI ~ 85%), which was resumed following treatment cessation, resulting in a 2-fold longer survival time (*p* < 0.001). The anti-tumor activity of FA-DCNPs was accompanied by an increase in tumor infiltrating CTLs, mature DCs and M1-TAMs, while the number of M2-TAMs declined. Consistently, FA-DCNPs treatment resulted in increased secretion of the immunostimulatory cytokines TNF, IFN-γ and IL-12, whereas secretion of the immunosuppressive IL-10 dropped significantly (*p* < 0.001). To further corroborate the activation of a robust immune response, FA-DCNPs treated mice were re-challenged with subcutaneously injected ID8 cells at a distant site, to mimic metastases. This distant tumor cell inoculation was rapidly infiltrated by CTLs and its growth was dramatically attenuated. FA-DCNPs demonstrated an effective and selective co-targeting of both the tumor cells and the TIME 363.

**Figure 5:**
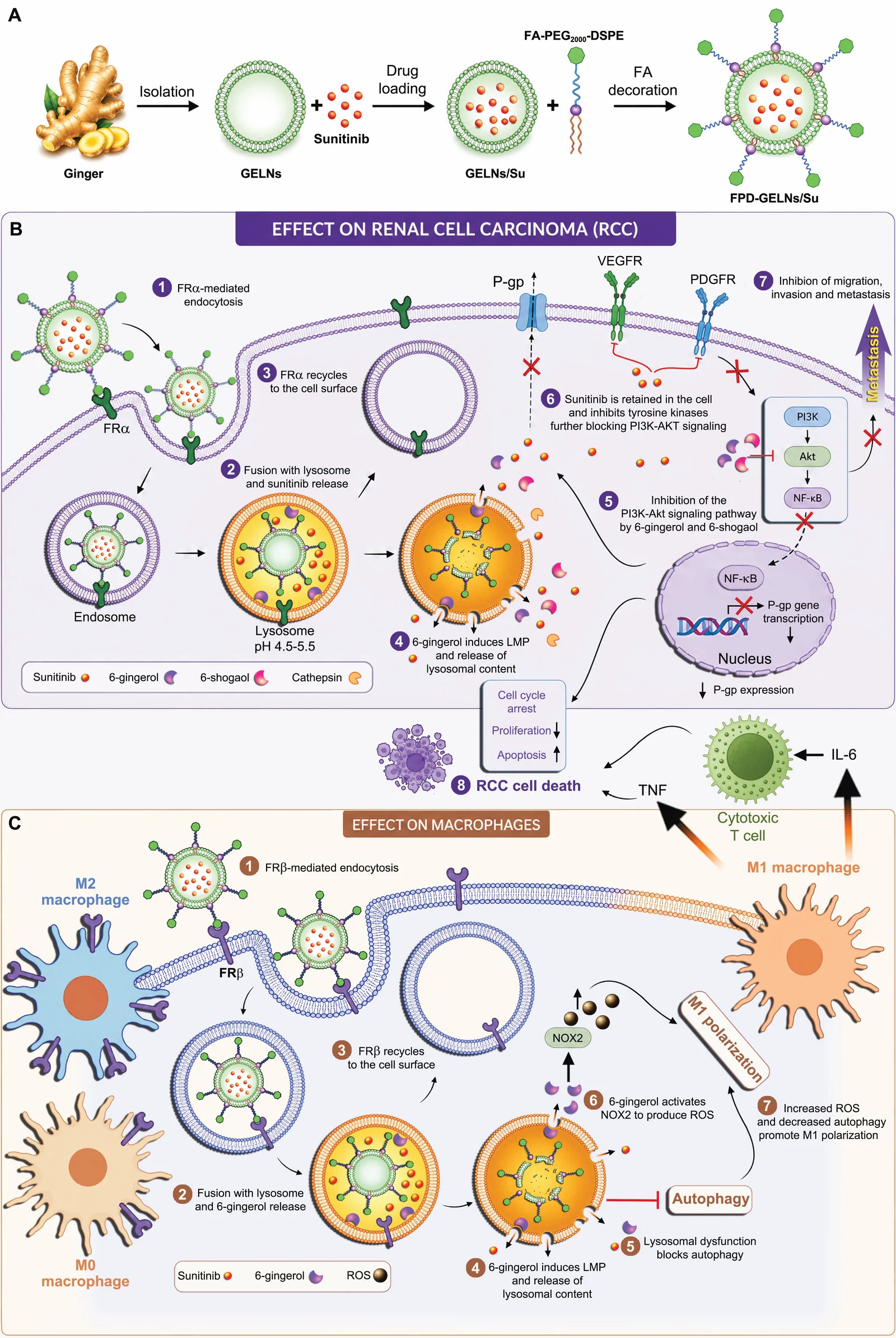
** Co-targeting tumor and immune cells by drug-loaded Ginger Exosome-Like Nanoparticles (GELNs).** A. Preparation of folic acid (FA)-decorated and sunitinib loaded GELNs (FPD-GELNs/Su). GELNs are isolated from fresh ginger homogenate by sucrose gradient centrifugation, and loaded with sunitinib, a hydrophobic weak-base tyrosine kinase inhibitor (TKI). Finally, the GELNs are decorated with FA-PEG. B. Induction of tumor cell death by FPD-GELNs/Su. 1) Renal cell carcinoma cells overexpress FRα, which binds FPD-GELNs/Su and mediates their endocytosis. 2) The endosomes fuse with lysosomes. Under the acidic lysosomal conditions, sunitinib and the ginger constituent 6-gingerol are released from the destabilized GELNs, while FA dissociates from FRα. 3) FRα recycles to the plasma membrane. 4) 6-gingerol induces lysosomal membrane permeabilization (LMP), allowing the release of the lysosomal content, including cathepsins, sunitinib, 6-gingerol and the ginger constituent 6-shogaol, into the cytosol. 5) 6-gingerol and 6-shogaol inhibit the PI3K-Akt signaling pathway, blocking the nuclear entrance of the downstream effector NF-κB. This leads to decreased transcription of NF-κB-responsive genes, including *ABCB1* which encodes the ATP-driven membrane multidrug exporter, P-glycoprotein (P-gp). 6) Following the decreased expression of P-gp, its substrate sunitinib is no longer extruded out of the cell and is retained at high intracellular concentrations. Sunitinib inhibits, amongst others, the receptor tyrosine kinases vascular endothelial growth factor receptor (VEGFR) and platelet-derived growth factor receptor (PDGFR), hence further blocking their downstream PI3K-Akt signaling pathway. 7) The potent inhibition of the PI3K-Akt signaling axis abolishes tumor cell migration and invasion, preventing them from metastasizing. 8) The combined effects of LMP and the blockade of the PI3K-Akt signaling pathway lead to cell cycle arrest, decreased proliferation and enhanced apoptosis of the tumor cells. C. Polarization of tumor-associated macrophages (TAMs) to the anti-tumor M1 phenotype. 1) The immunosuppressive M2, and to a lesser degree, M0 macrophages overexpress FRβ, which binds FPD-GELNs/Su and mediates their endocytosis. 2) The endosomes fuse with lysosomes. Under the acidic lysosomal conditions, sunitinib and the ginger constituent 6-gingerol are released from the destabilized GELNs, while FA dissociates from FRβ. 3) FRβ recycles to the plasma membrane. 4) 6-gingerol induces LMP, allowing the cytosolic release of the lysosomal content, including sunitinib and 6-gingerol. 5) Following LMP, lysosomal dysfunction blocks the progression of autophagy. 6) 6-gingerol activates NADPH oxidase 2 (NOX2) which induces the production of reactive oxygen species (ROS). 7) Decreased autophagy and increased ROS promote the polarization of M0 and M2 macrophages to the M1 phenotype. M1 macrophages secrete pro-inflammatory cytokines such as IL-6, which stimulates the activity and tumor infiltration of cytotoxic T cells, and tumor necrosis factor (TNF) which triggers tumor cell death. M1 macrophages also engulf and digest cancer cells via phagocytosis. The induction of LMP within macrophages, and the release of cathepsins, sunitinib and 6-gingerol can also induce macrophage cell death, which results in the release of ROS and cytokines into the tumor microenvironment (TME), thus inducing the polarization of other immunosuppressive cells.

An additional demonstration of FA-directed co-targeting NPs was published by Haoyu Xu *et al.,* where FA-PEG decorated ginger-derived exosome-like nanoparticles (GELNs) loaded with sunitinib (FPD-GELNs/Su) were used for the treatment of renal cell carcinoma (RCC) 376. Sunitinib is a multi-targeted TKI 377 used for the treatment of gastrointestinal and pancreatic neuroendocrine tumors as well as RCC 378. As a hydrophobic weak-base small-molecule, sunitinib highly accumulates in lysosomes and has strong natural fluorescence, traits that have been exploited for PDT 379-381. Sunitinib is a substrate of P-gp, and can be readily extruded from the cell by this ATP-driven efflux transporter 382,383. The biologically active constituents of ginger, mainly 6-gingerol and 6-shogaol, have been the subject of various studies as they were found to exert anticancer activities against gastrointestinal cancer 384,385. Isolated from fresh ginger homogenate by sucrose gradient centrifugation, GELNs are spherical bilayer membranous vesicles with a 100-200 nm diameter and a negative zeta-potential 376,386, which have been mainly studied as drug delivery systems 387,388. However, since the composition of GELNs also consists of biologically active components, they can have intrinsic anti-tumor capabilities. Indeed, before sunitinib loading, these GELNs exhibited a dose- and time-dependent cytotoxic activity against murine and human RCC cells, RenCa, OS-RC-2 and 786-O, with little effect against normal kidney HK-2 cells 376. In addition, GELNs inhibited colony formation, induced cell cycle arrest, and elicited anti-migratory and anti-invasive effects through inhibition of the PI3K-Akt signaling pathway 376, a mechanism previously suggested for both 6-gingerol 389 and 6-shogaol 390. In order for the GELNs to display significant anti-tumor activity *in vivo*, they had to be injected into the tumor, as any other administration resulted in their rapid depletion from the circulation and the gastrointestinal track 376. The addition of the FA-PEG coating increased the negative zeta potential of the NPs and greatly improved their biodistribution. This prevented the depletion of the NPs by spontaneous phagocytosis and allowed their intravenous injection. Following sunitinib loading, its release from the FPD-GELNs/Su was monitored. At physiological pH, the release of sunitinib occurred during the first 24 h and reached ~ 40%. At an acidic pH of 5.6, the release of sunitinib was greater and longer, achieving ~ 80% at 48 h. This suggests a good sunitinib release within lysosomes following RME. For compatibility with FPD-GELNs/Su, the expression levels of FRα and FRβ were quantified in the above cell lines. OS-RC-2 and HK-2 cells were negative for both receptors, while RenCa and 786-O cells expressed high levels of FRα 376. The expression of FRs in macrophage subtypes has been previously discussed. *In vitro* cytotoxicity assays at comparable concentrations of sunitinib, revealed that FPD-GELNs/Su had the lowest IC_50_ value in RenCa and 786-O cells, a value that was comparable to that of GELNs + free sunitinib. Although the fast diffusion rate of free sunitinib into cells far exceeds the slower internalization rates of sunitinib loaded NPs, treatment with either FPD-GELNs/Su or GELNs + free sunitinib resulted in higher cellular sunitinib concentrations than treatment with free sunitinib alone 376. Ginger-derived NPs have been shown to inhibit the PI3K/Akt/NF-κB signaling pathway and prevent the nuclear translocation of the central transcription factor NF-κB, thus attenuating transcription of various genes 391. Specifically, ferulic acid, which is one of the components of GELNs, inhibited NF-κB and reduced the expression of P-gp at the RNA and protein levels, thereby restoring drug sensitivity in drug resistant cells 392. In addition, 6-gingerol was shown to inhibit the efflux activity of P-gp and increase the cellular accumulation of P-gp substrate drugs 393. Hence, the authors explored the impact of GELNs on P-gp expression and found that FPD-GELNs/Su induced the strongest drop in P-gp mRNA and protein levels 376, suggesting a plausible mechanism for the enhanced sunitinib accumulation. The latter, along with the cytotoxic effect of the GELNs themselves, resulted in strong synergism between GELNs and free sunitinib, which was also found for FPD-GELNs/Su, i.e., combination index (CI) 394 of 0.1-0.3 376. One crucial aspect of the FPD-GELNs/Su system was overlooked by the authors and must be discussed. Owing to its physicochemical properties, following the RME of FPD-GELNs/Su, sunitinib should remain sequestered within the acidic lysosomes 395-397. Hence, we propose that GELNs destabilize the lysosomal membrane via 6-gingerol that has been shown to elicit LMP and cathepsin D release 398, thus allowing for the escape of sunitinib into the cytosol. In this respect, GELNs can also contribute to ICD through induction of LMP 200,399 and promote macrophage polarization to the M1 phenotype 147. Both M0 and M2 macrophages exhibited a ~ 68% internalization efficiency of GELNs within 24 h, while the addition of FA-PEG markedly enhanced their efficiency to 82% and 99%, respectively (*p* < 0.001) 376. FPD-GELNs also underwent a much more rapid internalization into M2 macrophages, surpassing 68% within 2 h. FPD-GELNs induced the polarization of M0 into M1 macrophages at comparable levels to the canonical LPS, and greatly surpassed it at converting M2 macrophages to the M1 phenotype. This conversion resulted, amongst others, in a >30-fold increase in IL-6 and a ~ 17-fold reduction in TGF-β (*p* < 0.001). Interestingly, 6-gingerol has been shown to induce the M2 to M1 polarization of macrophages in hepatocellular and lung carcinomas through the activation of NADPH oxidase 2 (NOX2) and production of ROS 400,401. The ability of FPD-GELNs to induce robust M1 polarization was recapitulated *in vivo* in RenCa-based xenograft mouse models. The increase in the M1:M2 ratio within the TAMs, induced by both FPD-GELNs and FPD-GELNs/Su, was accompanied by a 4.5-7.5-fold increase in tumor infiltrating CTLs, a ~ 2.5-fold increase in T helper cells (*p* < 0.01), and dramatic expression of IFNγ. Sunitinib had no significant effect on tumor growth, while FPD-GELNs exhibited strong TGI (*p* < 0.001), however, the synergistic effect of FPD-GELNs/Su induced excessive tumor cell apoptosis resulting in near complete tumor stasis (*p* < 0.001) 376. Blood tests and immunostaining revealed no drug related toxicity. Lastly, the ability of FPD-GELNs/Su to prevent RCC-related lung metastasis was also tested. Control mice developed ~ 100 lung nodules, a number that was not statistically different following sunitinib treatment, unless a much higher concentration was used. On the other hand, treatment with FPD-GELNs/Su decreased the number of metastatic lesions by ~ 80% 376. FPD-GELNs/Su represent a multi-mechanism and synergistic therapeutic approach for the selective co-targeting of tumor cells and their TIME (Figure 5).

## 6. Summary

In conclusion, the expression pattern of FRs makes them *bona fide* targets for the efficacious selective delivery of either cytotoxic drugs or immune modulating factors. In case of an FRα-positive tumor, the co-targeting of the tumor cells and the surrounding TIME by a FA-tagged lysosomal disruptive agent achieved excellent pre-clinical results and should be further developed. In case of an FRα-negative tumor, reprograming of the TIME by FRβ-targeted agents can be successfully combined with validated ICIs for enhanced invigoration of the immune system. While FRα-selective ADCs were developed for tumor targeting, the ability of ADCs to induce ICD also potentiates immune cells. Hence, future designs could benefit from the combination of a homogenous ADC with an LMP-inducing payload that would elicit ICD. With regards to ADCs, special attention should be given to the latest FR-targeted ADC AZD5335, which has shown promising initial results in PROC patients with low FRα expression. This ADC could represent a real breakthrough in the treatment of FRα-expressing tumors.

## Abbreviations

5-MTHF: 5-methyltetrahydrofolate
ADC: antibody-drug conjugate
ADCC: antibody-dependent cell-mediated cytotoxicity
ADCP: antibody-dependent cell-mediated phagocytosis
AIBW: adjusted ideal body weight
αKG: alpha ketoglutarate
ALK: anaplastic lymphoma kinase
AML: acute myeloid leukemia
APCs: antigen-presenting cells
α-SMA: alpha-smooth muscle actin
ATP: adenosine triphosphate
Atz: atezolizumab
BAT: basophil activation test
BCRP: breast cancer resistance protein, ABCG2
BET: bromodomain and extra-terminal
BRD3: bromodomain-containing protein 3
CAFs: cancer-associated fibroblasts
CARs: chimeric antigen receptors
CDC: complement-dependent cell death
CI: combination index
CR: complete response
CRT: calreticulin
CTLs: cytotoxic T-lymphocytes
CTLA-4: cytotoxic T lymphocyte-associated antigen-4
Ctx: Cetuximab
DAMP: damage-associated molecular pattern
DAR: drug to antibody ratio
DCs: dendritic cells
DM4: *N*^2′^-deacetyl-*N*^2′^-(4-mercapto-4-methyl-1-oxopentyl)-maytansine
DON: 6-diazo-5-oxo-l-norleucine
EGF: epidermal growth factor
EGFR: epidermal growth factor receptor
EMA: European Medicines Agency
FA: folic acid
Fab: fragment antigen-binding
FAP: fibroblast activation protein
FcγR: Fcγ receptor
FcRn: neonatal Fc receptor
FDA: US Food and Drug Administration
FFPE: formalin-fixed paraffin-embedded
FPGS: folylpoly-γ-glutamate synthetase
FR: folate receptor
FRTAC: folate receptor targeting chimera
GELNs: ginger-derived exosome-like nanoparticles
GM-CSF: granulocyte-macrophage colony-stimulating factor
GSH: glutathione
GPI: glycosyl-phosphatidylinositol
HER2: human epidermal growth factor receptor 2
HMGB1: high mobility group box 1
ICD: immunogenic cell death
ICIs: immune checkpoint inhibitors
IFNγ: interferon-gamma
IHC: immunohistochemistry
IL-4: interleukin-4
IMI: intraoperative molecular imaging
ITC: isothermal titration calorimetry
Lap: lapatinib
LMP: lysosome membrane permeabilization
LPS: lipopolysaccharide
mAbs: monoclonal antibodies
MAFs: metastasis-associated fibroblasts
M-CSF: macrophage colony stimulating factor
MDSC: myeloid-derived suppressor cells
MEK: Raf-activated MAP/ERK kinase
MIRV: mirvetuximab soravtansine-gynx, IMGN853, ELAHERE
MORAb-202: farletuzumab ecteribulin, farletuzumab-[Mal-PEG2-Val-Cit-PAB-eribulin]
MPRO: folate-PEG-S-S-PROTAC conjugate
MTA: microtubule targeting agent
MTD: maximum tolerated dose
MyD88: myeloid differentiation primary response 88
NF-κB: nuclear factor kappa-light-chain-enhancer of activated B cells
NIR: near infra-red
NK: natural killer
NOX2: NADPH oxidase 2
NPs: nanoparticles
NPSA: non-platinum single-agent
NSCLC: non-small cell lung cancer
OC: ovarian cancer
ORR: objective response rate
OS: overall survival
PARPi: poly ADP-ribose polymerase inhibitor
PBMCs: peripheral blood mononuclear cells
PCFT: proton-coupled folate transporter, SLC46A1
PD: progressive disease
PD-1: programmed cell death protein 1
PD-L1: programmed death-ligand 1
PDT: photodynamic therapy
PDXs: patient-derived xenografts
P-gp: P-glycoprotein, ABCB1
PLGA: poly D,L-lactide-co-glycolic acid
POI: protein of interest
PR: partial response
PROC: platinum-resistant epithelial ovarian cancer
PSOC: platinum-sensitive ovarian cancer
RCC: renal cell carcinoma
RD: residual disease
RFC: reduced folate carrier, SLC19A1
RME: receptor-mediated endocytosis
RP2D: recommended phase II dose
ROS: reactive oxygen species
SC209: 3-aminophenyl hemiasterlin
scFv: single-chain variable fragment
SCID: severe combined immunodeficiency
SD: stable disease
sFRα: soluble FRα
Stz: Sacituzumab
sulfo-SPDB: *N*-succinimidyl 4-(2-pyridyldithio)-2-sulfobutanoate
TAMs: tumor associated macrophages
TBR: tumor-to-background ratio
TEAEs: treatment emergent adverse events
TGF-α: transforming growth factor alpha
TGI: tumor growth inhibition
TIME: tumor immune microenvironment
TKIs: tyrosine kinase inhibitors
TLRs: toll-like receptors
TME: tumor microenvironment
TNF: tumor necrosis factor
TOP1i: topoisomerase-1 inhibitor
TROP2: trophoblast cell surface antigen-2
Ttz: trastuzumab
UPS: ubiquitin proteasome system
USC: uterine serous carcinoma
VATS: video-assisted thoracoscopic surgery
VEGF: vascular endothelial growth factor
VHL: Von Hippel-Lindau
VISTA: v-domain immunoglobulin suppressor of T-cell activation
